# A novel PD-L1-targeted shark V_NAR_ single-domain-based CAR-T cell strategy for treating breast cancer and liver cancer

**DOI:** 10.1016/j.omto.2022.02.015

**Published:** 2022-02-20

**Authors:** Dan Li, Hejiao English, Jessica Hong, Tianyuzhou Liang, Glenn Merlino, Chi-Ping Day, Mitchell Ho

**Affiliations:** 1 Laboratory of Molecular Biology, Center for Cancer Research, National Cancer Institute, Bethesda, MD 20892, USA; 2 Laboratory of Cancer Biology and Genetics, Center for Cancer Research, National Cancer Institute, Bethesda, MD 20892, USA

**Keywords:** shark V_NAR_, single-domain antibody, CAR-T cells, immune checkpoint, PD-L1, triple-negative breast cancer, hepatocellular carcinoma, liver cancer, glypican-3, GPC3

## Abstract

Chimeric antigen receptor (CAR)-T cell therapy shows excellent potency against hematological malignancies, but it remains challenging to treat solid tumors, mainly because of a lack of appropriate antigenic targets and an immunosuppressive tumor microenvironment (TME). The checkpoint molecule programmed death-ligand 1 (PD-L1) is widely overexpressed in multiple tumor types, and the programmed death-ligand 1 (PD-1)/PD-L1 interaction is a crucial mediator of immunosuppression in the TME. Here we constructed a semi-synthetic shark V_NAR_ phage library and isolated anti-PD-L1 single-domain antibodies. Among these V_NAR_s, B2 showed cross-reactivity to human, mouse, and canine PD-L1, and it partially blocked the interaction of human PD-1 with PD-L1. CAR (B2) T cells specifically lysed human breast cancer and liver cancer cells by targeting constitutive and inducible expression of PD-L1 and hindered tumor metastasis. Combination of PD-L1 CAR (B2) T cells with CAR T cells targeted by GPC3 (a liver cancer-specific antigen) regresses liver tumors in mice. We concluded that PD-L1-targeted shark V_NAR_ single-domain-based CAR-T therapy is a novel strategy to treat breast and liver cancer. This study provides a rationale for potential use of PD-L1 CAR-T cells as a monotherapy or in combination with a tumor-specific therapy in clinical studies.

## Graphical Abstract

**Figure:**
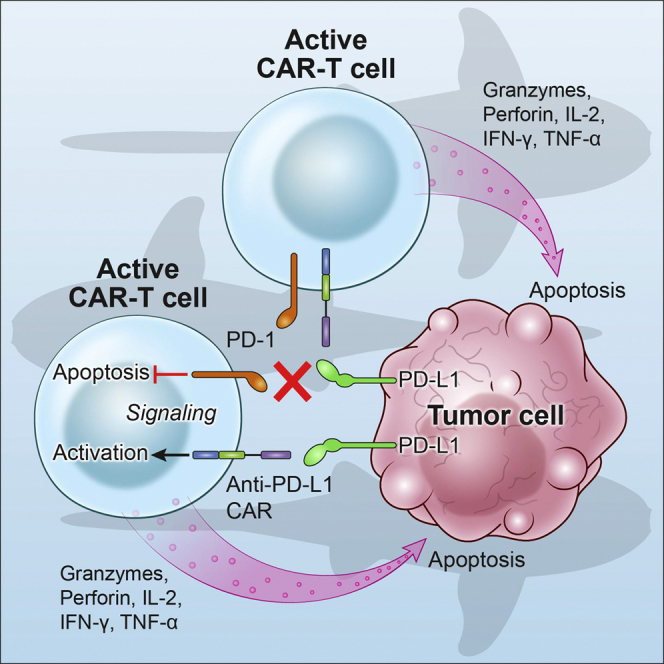

## Teaser

Li et al. constructed a novel semi-synthetic shark single-domain library by phage display and isolated a shark single-domain antibody specific for PD-L1. They incorporated the single-domain antibody in CAR-T cells that exhibited potent anti-tumor efficacy in mice bearing human breast cancer and liver cancer.

## Introduction

Adoptive cell therapy (ACT), particularly chimeric antigen receptor (CAR)-T cell therapy, has shown great potency as one of the most effective cancer immunotherapies.^1^, ^2^, ^3^ CARs are synthetic receptors consisting of an extracellular domain, a hinge region, a transmembrane domain, and intracellular signal domains (e.g., CD3-zeta, CD28, and 41BB) that initiate T cell activation.^4^, ^5^, ^6^ CARs can promote non-major histocompatibility complex (MHC)-restricted recognition of cell surface components, bind tumor antigens directly, and trigger a T cell anti-tumor response.^7^ So far, CAR-T cells targeting the B cell antigen CD19 have shown clinical success in individuals with advanced B cell lymphoma, which led to their approval by the US Food and Drug Administration (FDA).^3^^,^^8^ However, translation of CAR-T therapy to solid tumors is more difficult because of a lack of appropriate antigenic targets and the complex immunosuppressive tumor microenvironment (TME). Recently, the proteins glypican-2 (GPC2),^9^ glypican-3 (GPC3),^10^ and mesothelin^11^^,^^12^ have been reported as emerging antigens for CAR-T therapy for treatment of solid tumors and development for clinical trials. However, not all tumors express particular surface antigens suitable for CAR recognition. Tumor heterogeneity makes targeted therapy more challenging. Programmed death-ligand 1 (PD-L1 or CD274) has aberrantly high expression on multiple tumor types through oncogenic signaling^13^ and is induced by pro-inflammatory factors such as interferon (IFN)-γ in the immunoreactive TME.^14^ It has been shown that PD-L1 expressed on tumors can induce T cell tolerance and avoid immune destruction through binding with its ligand programmed cell death protein 1 (PD-1) on T cells, which may be one of the main reasons for the poor effect of CAR-T cells in solid tumors.^15^ Clinically, antibody-based PD-1/PD-L1 antagonists have been reported to induce durable tumor inhibition, especially in melanoma, non-small cell lung cancer, and renal cancer. However, the response rate remains poor in other types of advanced solid tumors.^16^ PD-L1-targeting camelid V_H_H-nanobody-based CAR-T cells have been shown to delay tumor growth in a syngeneic mouse melanoma model.^17^

PD-L1-targeting CAR natural killer (NK) cells inhibit growth of triple-negative breast cancer (TNBC), lung cancer, and bladder tumors engrafted in non-obese diabetic (NOD) severe combined immunodeficiency (SCID) gamma (NSG) mice.^18^ Bispecific Trop2/PD-L1 CAR-T cells targeting Trop2 and PD-L1 demonstrate the improved killing effect of CAR-T cells in gastric cancer.^19^ PD-L1-targeted CAR-T cell therapy is presumed to kill PD-L1-overexpressing tumor cells and block the PD-1/PD-L1 immune checkpoint, significantly enhancing anti-tumor activity in solid tumors.

Single-chain variable fragment (scFv), a type of recombinant antibody, commonly serves as the antigen recognition region of a CAR construct. It consists of variable heavy (V_H_) and variable light (V_L_) chains connected by a flexible linker (Gly_4_Ser)_3_. However, folding of an artificially engineered scFv can affect the specificity and affinity of the CAR for its target antigen.^20^ Alternatively, the antigen-binding domain of naturally occurring single-domain antibodies (heavy chain only) from camelids (V_H_H)^21^ and sharks (V_NAR_)^22^ have beneficial properties for engineering of CARs. They are small (12–15 kDa), easy to express, and capable of binding concave and hidden epitopes inaccessible to conventional antibodies.^23^ Remarkably, shark V_NAR_s have unique features distinct from camel V_H_Hs—they have great diversity and are evolutionarily derived from an ancient single domain that functions as a variable domain in B cell and T cell receptors.^24^^,^^25^ We previously constructed a V_NAR_ phage display library from six nurse sharks.^26^ Currently, several shark V_NAR_s are in pre-clinical research, and their therapeutic and biotechnological applications are under intensive investigation.^27^, ^28^, ^29^ In this study, to improve the diversity of the shark V_NAR_ repertoire, we constructed a semi-synthetic shark V_NAR_ phage library with randomized third complementarity-determining regions (CDR3s) 18 amino acids (aa) in length. Of the three cross-reactive binders, only V_NAR_ B2 could functionally block the interaction between human PD-L1 and PD-1. More importantly, B2-based CAR-T cells successfully inhibited tumor growth in xenograft mouse models of TNBC and hepatocellular carcinoma (HCC). Interestingly, the combination of anti-PD-L1 CAR (B2)-T cells and anti-GPC3 CAR-T cells synergistically demonstrated better efficacy than single-antigen-targeted CAR-T cells in the HCC xenograft mouse model, highlighting the feasibility and efficacy of shark V_NAR_-based CAR-T cells targeting PD-L1 in solid tumors.

## Results

### Construction of a semi-synthetic shark V_NAR_ single-domain library

**Figure 1:**
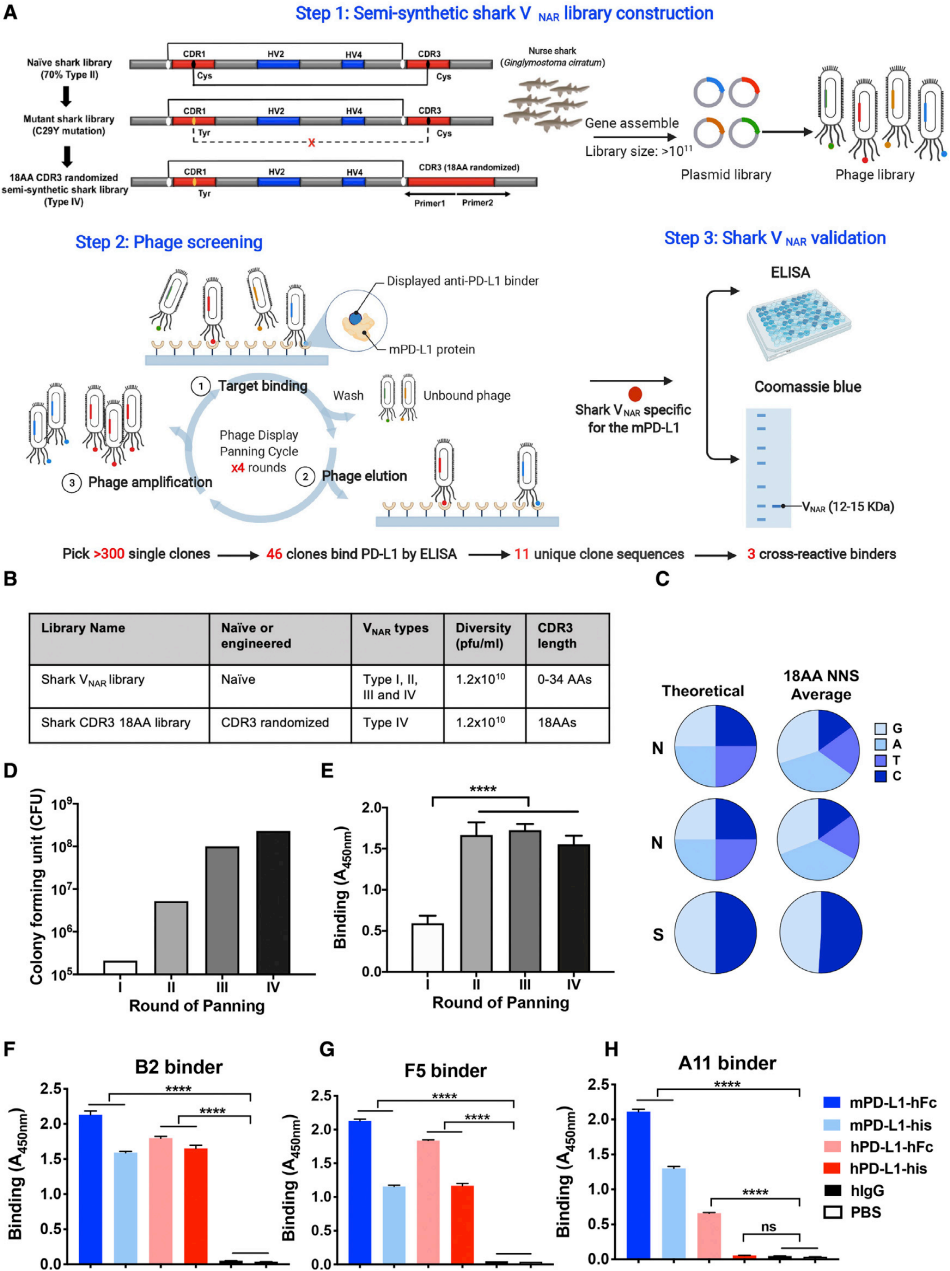
Isolation of the anti-PD-L1 single-domain antibody by phage display from an engineered semi-synthetic shark V_NAR_ phage library (A) Circuit of three steps of library construction and phage panning. An 18-aa randomized CDR3 semi-synthetic shark V_NAR_ phage library was constructed by PCR mutation and gene assembly. After 3–5 rounds of phage panning, anti-mPD-L1 V_NAR_s were isolated from the phage library and validated by phage ELISA and protein purification technologies. (B) Information regarding the new shark V_NAR_ library compared with the pre-synthetic V_NAR_ library. (C) Pie chart of the percentages of average nucleotide (ACTG) ratio at each randomization NNS (where N = A/C/G/T, and S = C/G). (D) Phage-displayed single-domain antibody clones were identified against recombinant mPD-L1-His after four rounds of panning. A gradual increase in phage titers was observed during each round of panning. (E) Polyclonal phage ELISA from the output phage of each round of panning. (F–H) Cross-reactivity of anti-PD-L1 B2 (F), A11 (G), and F5 (H) to mPD-L1 and hPD-L1 protein within the His tag or hFc tag by monoclonal phage ELISA.

We previously constructed a naive shark V_NAR_ library from 6 naive adult nurse sharks (*Ginglymostoma cirratum*) with a size of 1.2 × 10^10^ plaque-forming units (PFUs)/mL.^25^^,^^26^ To improve the diversity and utility of the shark V_NAR_ library, here we developed a semi-synthetic randomized CDR3 shark V_NAR_ library (referred to as the ’18-aa CDR3 shark library). As illustrated in Figure 1A, 70% of V_NAR_s in the naive shark library are type II, containing two canonical cysteines located at aa 21 and 82 to form a disulfide bond and at least one extra cysteine in CDR1 and CDR3 to form an interloop disulfide bond. Because the type IV V_NAR_ sequence is the closest to its mammalian counterpart, such as human V_H_ with only a pair of canonical cysteines (one before CDR1 and the other before CDR3), we mutated C29Y in CDR1 and randomized the CDR3 loop region to change all four types (type I, II, III, and IV) to type IV V_NAR_s. The diversity of the new semi-synthetic library is approximately 1.2 × 10^10^ PFUs/mL, which is comparable with our original naive shark V_NAR_ library (Figures 1A and 1B). To assess the randomness of sequence modification, we estimated the average nucleotide ratio at each CDR3 residue based on sequencing analysis and found that the CDR3 nucleotides were completely randomized with the desired ATGC bases ratios (Figure 1C).

### Isolation of cross-species V_NAR_ single domains with high affinity for PD-L1

To identify the anti-PD-L1 shark V_NAR_ that functions in the murine tumor environment, we used mouse PD-L1 (mPD-L1) protein as an antigen to screen the new semi-synthetic shark library (Figure 1A). After four rounds of panning, ≈1,000-fold enrichment of eluted phage colonies was obtained (Figure 1D). We also observed enhanced binding to PD-L1 after the first round of phage panning (Figure 1E). At the end of the fourth round of panning, 46 individual clones were identified to bind mPD-L1 protein by the monoclonal phage enzyme-linked immunosorbent assay (ELISA), and 11 unique binders were confirmed by subsequent sequencing. Three PD-L1-specific V_NAR_s (B2, A11, and F5) finally showed cross-reactivity to mPD-L1 and human PD-L1 (hPD-L1) protein in the His tag or hFc tag format, as shown by monoclonal phage ELISA (Figures 1F–1H).

**Figure 2:**
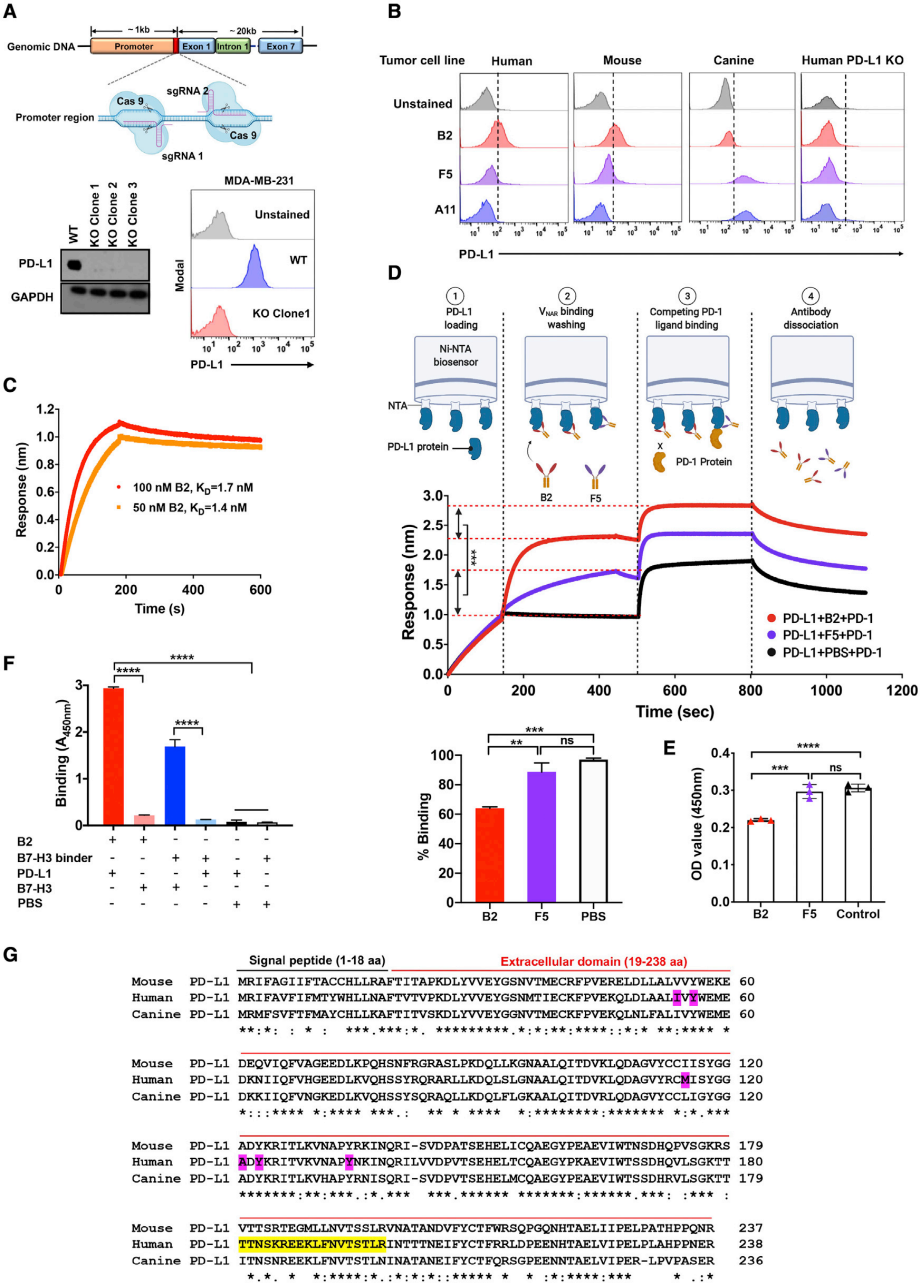
Verification of the specific binding and blocking ability of anti-PD-L1 shark V_NAR_s (A) Schematic of constructing the PD-L1 KO MDA-MB-231 cell line using CRISPR-Cas9. Two sgRNAs were designed to target the promoter of the endogenous PD-L1 gene. Single PD-L1 KO clones were validated by western blot and flow cytometry. (B) The cross-reactive binding of anti-PD-L1 V_NAR_s to native PD-L1 as determined by flow cytometry. Three different tumor cell lines (the human breast cancer cell line MDA-MB-231, murine melanoma cell line B8979HC, and canine tumor cell line Jones) were stained with V_NAR_s. (C) Binding kinetics of B2-hFc to hPD-L1 protein. (D-E) Blocking activity of V_NAR_-hFc to the interaction of hPD-L1 and hPD-1 as determined by the Octet platform (D) and sandwich ELISA (E). (F) Specific binding of B2 to hPD-L1 and hB7-H3. (G) Epitope mapping of individual B2, F5, and A11 and sequence alignment of the PD-L1 ECD region of human, mouse, and dog. Conserved residues are marked with asterisks, residues with similar properties between variants are marked with colons, and residues with marginally similar properties are marked with periods. The main binding residues of the hPD-L1 identified previously that interact with PD-1 are shaded in magenta. The binding peptides of B2 to hPD-L1 are highlighted in yellow. Values represent mean ± SEM. ∗∗p < 0.01; ∗∗∗p < 0.001; ∗∗∗∗p < 0.0001; ns, not significant.

To determine the antigen specificity of shark V_NAR_s, we established PD-L1 knockout (KO) single clones by CRISPR-Cas9 technology in a human TNBC cell line, MDA-MB-231. To enhance the PD-L1 KO efficiency, we designed two single guide RNAs (sgRNAs) targeting the promoter of the endogenous PD-L1 gene (Figure 2A). All three individual cell clones were confirmed by loss of PD-L1 expression (Figure 2A), and clone 1 was used further in the present study. To determine the cross-species reactivity of anti-PD-L1 shark V_NAR_s against native PD-L1, three PD-L1-positive tumor cell lines, including a human breast cancer cell line, a mouse melanoma cell line, and a canine melanoma cell line, were used to evaluate the binding ability of B2, A11, and F5. As shown in Figure 2B, B2 and F5 bind human antigens and cross-react with mouse and canine antigens. B2 showed a higher binding ability to human and mouse antigens than F5. A11 binds canine antigen but not human or mouse antigen. In contrast, no binding was shown on PD-L1 KO cells, indicating that the binding ability of shark V_NAR_s is antigen specific.

We further produced V_NAR_-hFc fusion proteins and incubated them with hPD-L1-His protein on the biolayer interferometry (BLI) Octet platform to determine binding kinetics. The K_D_ value of B2 was 1.7 nM and 1.4 nM at a concentration of 100 nM and 50 nM, respectively (Figure 2C), whereas F5 failed to yield an accurate K_D_ value because it showed slight non-specific binding to the nickel-charged tris-nitrilotriacetic acid (Ni-NTA) sensor on Octet. To examine whether B2 could functionally block the interaction between human PD-1 (hPD-1) and hPD-L1, we developed a blocking assay based on BLI technology. As shown in Figure 2D, B2 partially blocked the interaction of hPD-1 with hPD-L1 compared with the PBS control. In contrast, F5 showed positive binding to hPD-L1 but could not block the hPD-1/hPD-L1 interaction. We conducted sandwich ELISA to validate the functional blocking capacity of B2. It showed that the V_NAR_ did partially block the interaction of hPD-1 with hPD-L1 (Figure 2E). In addition, V_NAR_ B2 specifically binds to hPD-L1 but not human B7-H3, another B7-CD28 family member (Figure 2F).

To identify the binding epitope of anti-PD-L1 nanobodies, we synthesized a peptides array based on the hPD-L1 extracellular domain (ECD) consisting of a total of 24 peptides. As shown in Figures S1 and 2G, F5 and B2 strongly bind to peptide 19 (TTNSKREEKLFNVTSTLR), whereas A11 did not bind to any peptides.

We successfully identified functionally cross-species anti-PD-L1 shark V_NAR_ with high affinity.

### Anti-PD-L1 CAR (B2) T cells kill breast cancer cells

**Figure 3:**
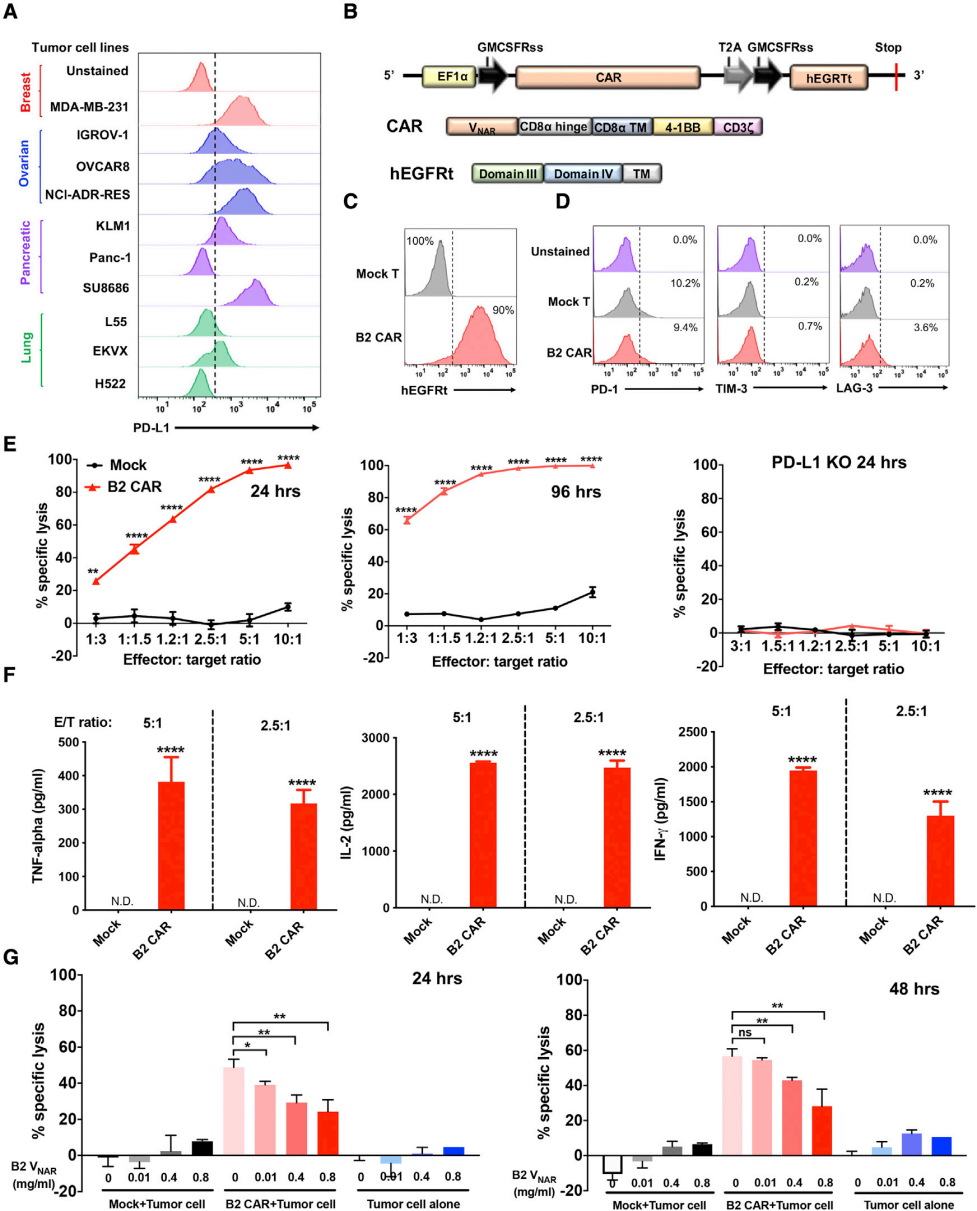
PD-L1 specific V_NAR_-based CAR-T cells exhibit antigen specific cytotoxicity against MDA-MB-231 (A) Surface PD-L1 expression on multiple human tumor types as determined by flow cytometry. (B) Construct of CAR (B2) T cells, where CAR and hEGFRt are expressed separately by the self-cleaving T2A ribosomal skipping sequence. (C) The transduction efficiency of CAR (B2) in T cells was determined by hEGFRt expression. Non-transduced T cells were the mock control. (D) Exhaustion marker expression on *in-vitro-*cultured mock T cell and CAR (B2) T cell populations. (E) Cytolytic activity of CAR (B2) T cells after 24 or 96 h of incubation with MDA-MB-231 GL or PD-L1 KO MDA-MB-231 GL, respectively, in a 2-fold dose-dependent manner. (F) TNF-α, IL-2, and IFN-γ concentrations in the supernatants of the killing assay at E/T ratios of 5:1 and 2.5:1 in (D), as measured by ELISA. (G) The monovalent B2 V_NAR_ protein specifically inhibited the cytotoxicity of CAR (B2) T cells on MDA-MB-231 cells after 24- and 48-h incubation. Statistical analyses are shown from three independent experiments. Values represent mean ± SEM. ∗∗p < 0.01; ∗∗∗p < 0.001; ∗∗∗∗p < 0.0001.

Based on flow cytometry analysis, we found that PD-L1 was highly expressed in multiple human tumor types, including breast cancer (MDA-MB-231), ovarian cancer (IGROV-1, OVCAR8, and NCI-ADR-RES), pancreatic cancer (KLM1 and SU8686), and lung cancer (EKVX), suggesting that PD-L1 is a putative pan-cancer antigen (Figure 3A). To determine the application of our shark V_NAR_s to a CAR-T cell therapeutic approach, we constructed CARs containing the B2 V_NAR_ fragment as the antigen recognition region, along with 4-1BB, CD3ζ signaling domains, and a truncated human epidermal growth factor receptor (hEGFRt) cassette to gauge transduction efficiency and to switch off CAR (Figure 3B). The transduction efficiency of CAR (B2) T was high (∼90%) (Figure 3C). During days 7–12, non-transduced mock T and CAR (B2) T cells showed indistinguishable expression of exhaustion markers (PD-1 and TIM-3) compared with each other, whereas a slightly higher expression of LAG-3 was found in CAR (B2) T cells compared with mock T cells (Figure 3D). MDA-MB-231 is a highly aggressive, invasive, and poorly differentiated TNBC cell line with limited treatment options. We therefore engineered it to overexpress GFP/luciferase (GL) to establish a luciferase-based cytolytic assay. Mock T and CAR (B2) T cells were incubated with MDA-MB-231 GL cells for 24 or 96 h. As shown in Figure 3E, CAR (B2) T cells effectively lysed tumor cells in a 2-fold dose-dependent manner compared with mock T cells. Moreover, the 96-h incubation procedure improved the cytotoxicity of CAR (B2) T cells even at the lowest effector:target (E/T) ratio of 1:3. To investigate whether the cytolytic activity of CAR (B2) T cells is antigen dependent, we incubated CAR (B2) T cells with MDA-MB-231 PD-L1 KO cells. This showed that CAR-T cells could not kill antigen KO cells (Figure 3E). A dramatically higher level of tumor necrosis factor alpha (TNF-α), interleukin-2 (IL-2), and IFN-γ was released from CAR T cells at E/T ratios of 5:1 and 2.5:1, whereas there was minimum cytokine production in mock T cells (Figure 3F). These results suggested that V_NAR_-based CAR-T cells could lyse tumor cells by efficiently targeting PD-L1. We included a corresponding soluble B2 V_NAR_ in the co-culture system to detect whether it could affect the cytotoxicity of CAR (B2) T cells by competitively blocking the recognition site on tumor cells. As shown in Figure 3G, inclusion of the B2 single domain significantly inhibited the cytolytic activity of CAR (B2) T cells. In contrast, no specific lysis in tumor cells was found in coincubation with mock T cells or tumor cells alone in the presence of B2. We concluded that CAR (B2) T cells could specifically lyse PD-L1-positive human tumor cells.

### Anti-PD-L1 CAR (B2) T cells inhibit orthotopic breast cancer growth in mice

**Figure 4:**
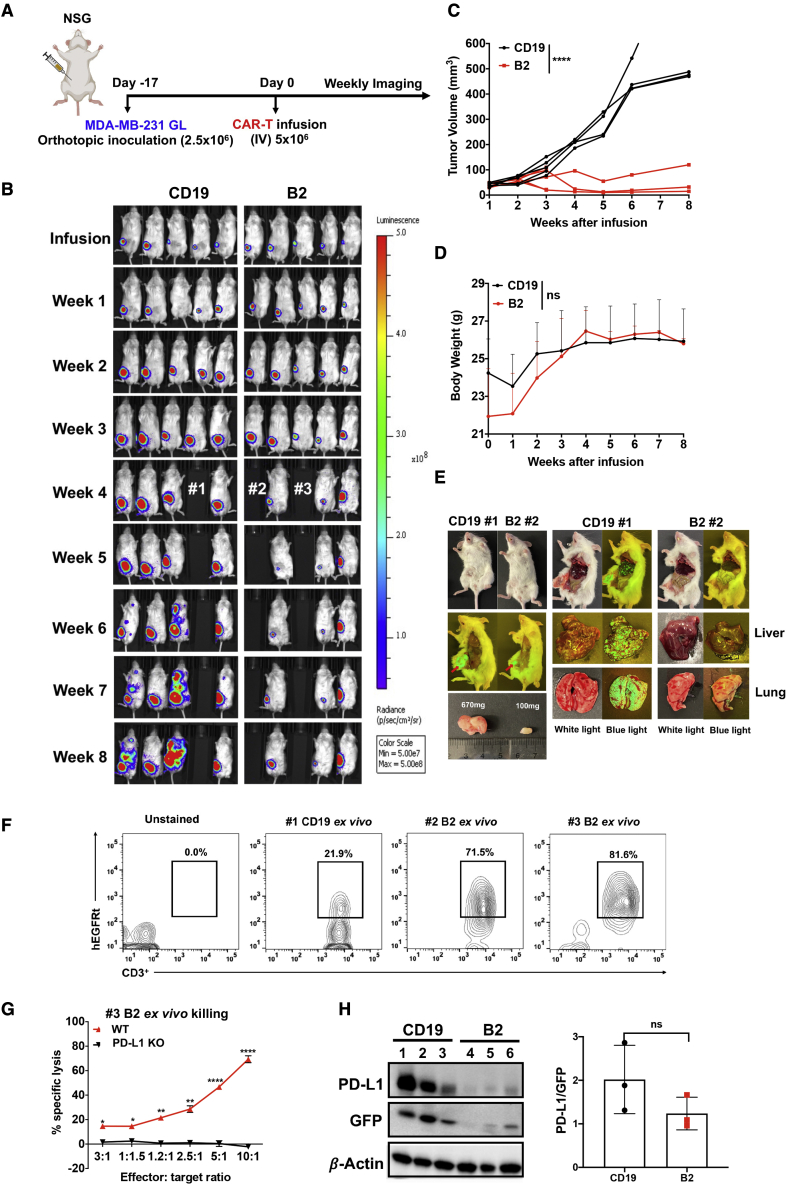
Tumor regression in the orthotopic MDA-MB-231 xenograft mouse model by anti-PD-L1 CAR (B2) T cell infusion (A) Schematic of the MDA-MB-231 orthotopic xenograft NSG model infused i.v. with five million CAR (B2) T cells and CAR (CD19) T cells after 17 days of tumor inoculation. (B) Representative bioluminescence image of MDA-MB-231 tumor growth in the orthotopic model. (C) Tumor size of every mouse measured by a digital caliper [V = 1/2(length width^2^)]. ∗∗∗∗p < 0.0001. (D) Body weight of mice. Values represent mean ± SEM. (E) Representative pictures showing the restriction of tumor metastasis in CAR (B2) T cell-infused mice. (F-G) The persentage of persistent hEGFRt^+^ CAR-T cells in the total CD3^+^ human T cells recovered from mice after 3 weeks of CAR-T cell infusion (F) and their *ex vivo* killing on MDA-MB-231 tumor cells (G). (H) Detection of PD-L1 expression in MDA-MB-231 tumor xenografts by western blotting.

To evaluate the anti-tumor efficacy of PD-L1-specific CAR-T cells in mice, we established an orthotopic breast tumor xenograft model by implanting MDA-MB-231 GL cells into the fourth mouse mammary fat pad. Seventeen days after tumor inoculation, mice were infused intravenously (i.v.) with CAR (B2) T cells or antigen-mismatched CAR (CD19) T cells (Figure 4A). We used bioluminescence intensity and tumor volume to track the anti-tumor efficacy of CAR-T cells. At weeks 3–4 after treatment, we found that the tumor bioluminescence signal was saturated, as measured by the IVIS imaging system, suggesting that bioluminescence imaging might not be a suitable method to accurately measure tumor size, in particular for large tumors. Mice were followed up to 8 weeks after CAR-T cell infusion. We euthanized one CAR (CD19) T cell-treated mouse (mouse 1) and two CAR (B2) T cell-treated mice (mice 2 and 3) after week 3 to perform CAR-T cell persistence analysis (Figure 4B). We observed that CAR (B2) T cells dramatically reduced the breast tumor burden (Figures 4B and 4C) without a marked loss of body weight (Figure 4D). Tumors metastasized in control group mice after 5 weeks of CAR (CD19) T cell infusion (Figures 4B and 4E). We did not observe tumor metastases in mouse liver or lungs with CAR (B2) T cell infusion (Figures 4B and 4E). These data indicated that CAR (B2) T cells could treat breast cancer metastatic lesions. To determine CAR-T cell persistence, we recovered CAR (CD19) and CAR (B2) T cells from mouse spleen (mice 1, 2, and 3) at week 4. We found that *ex vivo* CAR (B2) T cells recovered from mice had a comparable persistence after 3 weeks of infusion (Figure 4F). These spleen-isolated CAR (B2) T cells still exhibited significant *ex vivo* cytotoxicity against PD-L1-positive tumor cells (wild type [WT]) compared with PD-L1 KO cells (Figure 4G), which suggested that these *in vivo* persistent CAR (B2) T cells remained robust. By the end of week 8, mice were euthanized, and tumors were isolated from 6 mice to analyze antigen expression after CAR-T cell treatment *in vivo*. We normalized PD-L1 expression by tumor-specific GFP expression and found that there was no significant difference in PD-L1 expression between the CAR (CD19) T cell group and CAR (B2) T cell group (Figure 4H).

### CAR (B2) T cells kill liver cancer cells by targeting inducible PD-L1

**Figure 5:**
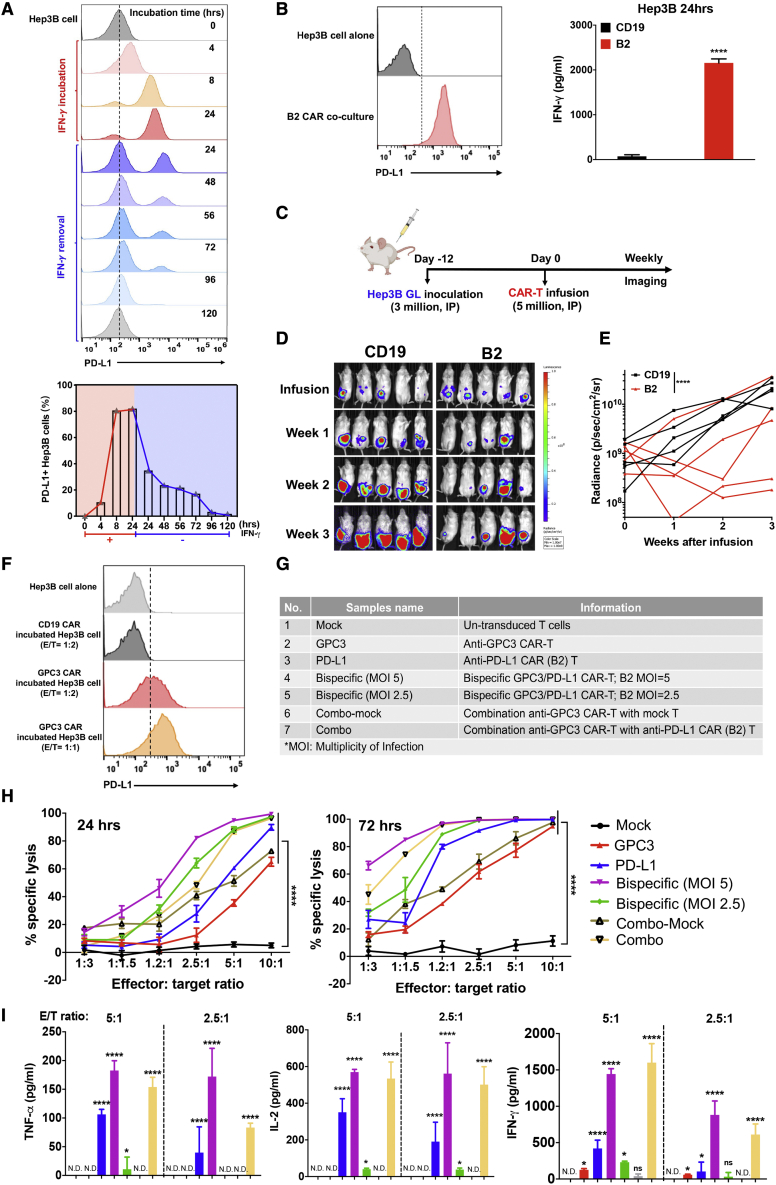
CAR (B2) T cells lysed Hep3B tumor by targeting inducible PD-L1 and improved CAR-T efficacy in bispecific CAR and combination manners (A) Inducible PD-L1 expression in Hep3B cells upon 50 μg/mL IFN-γ stimulation followed by depletion of IFN-γ at 24 h. (B) Inducible PD-L1 expression in the Hep3B cells after 24-h incubation with CAR (B2) T cells at an E/T ratio of 1:2. Shown are IFN-γ levels in cell supernatants of CAR (CD19) T cells or CAR (B2) T cells co-cultured with Hep3B cells. (C) Schematic of the Hep3B xenograft NSG model infused i.p. with five million CAR (B2) T cells and CAR (CD19) T cells after 12 days of tumor inoculation. (D) Representative bioluminescence image of Hep3B tumor growth in the xenograft model. (E) Tumor bioluminescence growth curve. (F) Inducible PD-L1 expression in Hep3B cells co-cultured with GPC3 CAR-T cells at an E/T ratio of 1:2 or 1:1 for 24 h. (G) Strategy of bispecific CAR-T cells and combination CAR-T cells targeting GPC3 and PD-L1. (H) Cytolytic activity of CAR-T cells on Hep3B cells after 24- or 72-h incubation *in vitro*. (I) TNF-α, IL-2, and IFN-γ concentration in the co-culture supernatant from (H) as measured by ELISA. Values represent mean ± SEM. ∗∗p < 0.01; ∗∗∗p < 0.001; ∗∗∗∗p < 0.0001.

We found inducible but not constitutive expression of PD-L1 in the liver cancer cell line Hep3B upon IFN-γ stimulation (Figure 5A). PD-L1 expression in Hep3B cells increased within a short period of IFN-γ incubation (4 h) and reached a peak at 8 h. Inducible PD-L1 expression decreased slowly and gradually over time after IFN-γ removal but remained for up to 96 h. These data suggested that PD-L1 expression in Hep3B cells could be up-regulated quickly and sustained for a relatively long time upon IFN-γ stimulation, suitable for PD-L1 CAR-T cell recognition and function. We also found inducible PD-L1 expression in Hep3B tumor cells co-cultured with CAR (B2) T cells for 24 h, which may in no small part be due to release of massive IFN-γ from CAR-T cells (Figure 5B). To test the anti-tumor effect of CAR (B2) T cells by targeting inducible PD-L1 *in vivo*, we established a xenograft mouse model with intraperitoneal (i.p.) injection of Hep3B GL tumor cells. After 12 days of tumor inoculation, mice were infused i.p. with CAR-T cells (Figure 5C). Four of 5 CAR (B2) T cell-treated mice showed a significant decrease in tumor growth compared with the control CAR (CD19) T cell group after 3 weeks of infusion (Figures 5D and 5E). Based on this observation, we believe that CAR (B2) T cells might benefit liver cancer therapy.

### Anti-PD-L1 CAR-T cells improve the killing effect of anti-GPC3 CAR-T cells *in vitro*

Our previous study developed GPC3-targeted CAR-T cells as an emerging liver cancer therapy.^10^ We observed that CAR (GPC3) T cells killed Hep3B tumor cells efficiently but inducible PD-L1 expression was found in the Hep3B cells co-cultured with CAR (GPC3) T cells rather than control CAR (CD19) T cells (Figure 5F), which may allow cancers to evade the host immune system. Therefore, we hypothesized that eliminating inducible PD-L1-positive tumor cells by CAR (B2) T cells in the TME could improve the efficacy of CAR-T cells targeting liver cancer. To test our hypothesis, we designed two strategies: bispecific CAR-T cells targeting PD-L1 and GPC3 and combination (Figure 5G). We produced bispecific CAR-T cells by co-transduction with a CAR (GPC3) and CAR (B2) lentivirus (Figure 5G). The transduction efficiency of bispecific CAR was similar to PD-L1 or GPC3 CAR based on hEGFRt expression (Figure S2A). We found that bispecific CAR had slightly weaker binding to PD-L1-hFc than PD-L1 CAR (21.9% versus 28.3%), whereas its binding ability to GPC3-hFc was similar to that of GPC3 CAR (17.3% versus 17.9%) (Figure S2B). To compare their anti-tumor effects, we incubated all seven groups of CAR-T cells and mock T cells with Hep3B cells for 24 and 72 h (Figure 5G). As shown in Figure 5H, the cytotoxicity of bispecific CAR was significantly higher than either of the monospecific CAR, especially at 72 h of incubation time. PD-L1 CAR-T cells could improve the efficiency of GPC3 CAR-T cells in a dose-dependent manner (MOI 2.5 versus 5).

The bispecific and combination CAR-T cell strategies showed dramatically higher TNF-α, IL-2, and IFN-γ levels than monospecific CAR-T cells upon tumor cell stimulation (Figure 5I). Therefore, we concluded that the bispecific CAR-T cell and combined CAR-T cell strategies significantly improved the activity of CAR-T cells in liver cancer by targeting PD-L1 and GPC3.

### Combination of PD-L1 CAR-T cells and GPC3 CAR-T cells achieves a synergistic anti-tumor effect in mice

**Figure 6:**
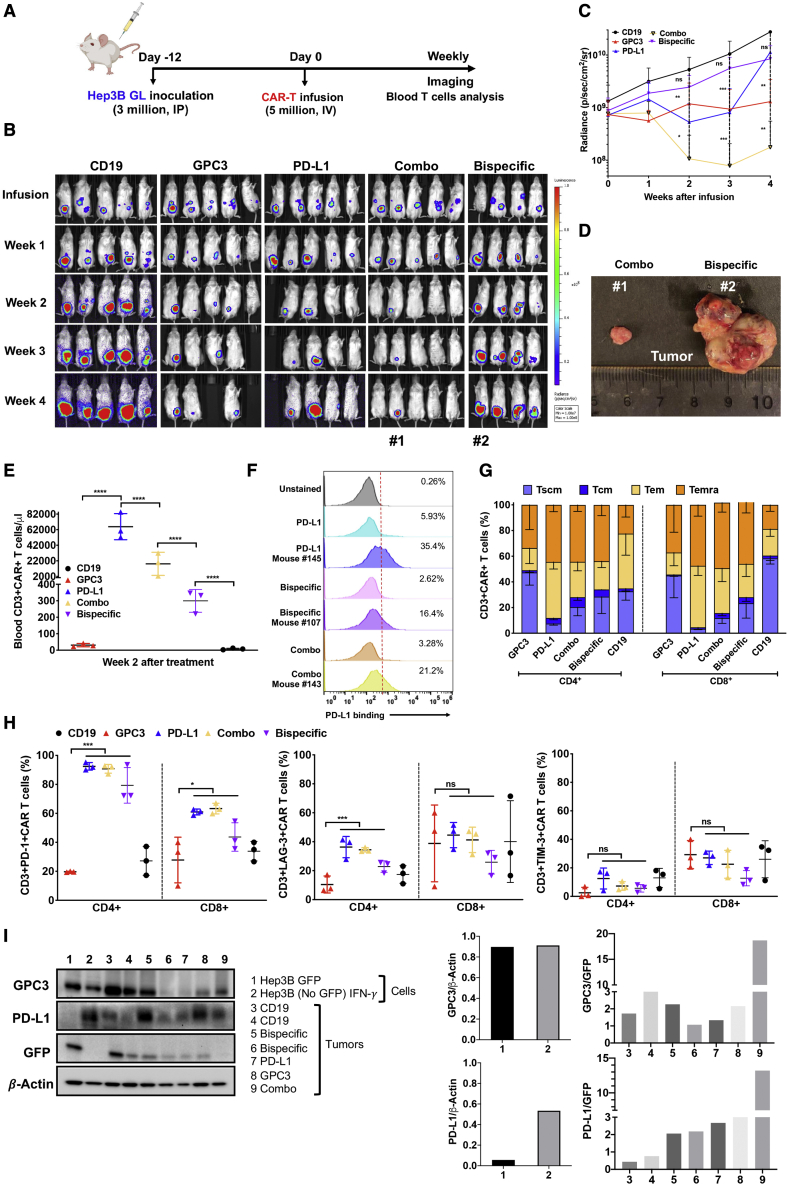
Combination of PD-L1 CAR-T cells and GPC3 CAR-T cells achieved a synergistic anti-tumor effect *in vivo* (A) Schematic of the Hep3B xenograft NSG model infused i.p. with an equivalent of a total of five million CAR-T cells after 12 days of tumor inoculation. (B) Representative bioluminescence image of Hep3B tumor growth in the xenograft model. (C) Tumor bioluminescence growth curve. (D) At the end of the study, the sizes of tumors in mice from the combination CAR group (mouse 1 mouse) and bispecific group (mouse 2). (E) Absolute CAR-T cell count detected in mouse peripheral blood after 2 weeks of treatment and absolute CAR-T concentration (cells per microliter) ±SD for all evaluated mice in each treatment group. (F) The binding ability of CAR T cells recovered *in vitro* and *in vivo* (2 weeks after treatment) to PD-L1 antigen using flow cytometry. (G) The relative proportion of stem cell-like memory (T_SCM_), central memory (T_CM_), effector memory (T_EM_), and terminally differentiated effector memory (T_EMRA_) cell subsets defined by CD62L, CD45RA, and CD95 expression in CD4^+^ and CD8^+^ CAR^+^ T cell population in mouse blood at week 2 of treatment. (H) Exhaustion marker expression on CD4^+^ and CD8^+^ CAR^+^ T cell populations in mouse blood at week 2 after treatment. (I) Western blotting detects GPC3 and PD-L1 expression in Hep3B tumor xenografts. The expression of GPC3 and PD-L1 in Hep3B GFP (no. 1) and Hep3B (no GFP) IFN-γ cells (no. 2) was normalized by β-actin. The expresssion of GPC3 and PD-L1 in tumor samples (no. 3–9) was nomalized by tumor-specific GFP expression.

To further analyze the functions of bispecific and combination CAR-T cell strategies in response to liver cancer *in vivo*, we established a Hep3B xenograft mouse model. Mice bearing Hep3B tumors were divided into five groups and infused with five million equivalents of CD19 CAR-T cells, GPC3 CAR-T cells, PD-L1 CAR-T cells, bispecific CAR-T cells, and a combination of 2.5 million GPC3 CAR-T cells and PD-L1 CAR-T cells (referred to as “Combo”), respectively. The tumor luciferase signal was evaluated by bioluminescence imaging weekly, and T cells isolated from week 2 mouse blood were analyzed by flow cytometry (Figure 6A). Compared with CD19 CAR-T cells, monospecific therapy of GPC3 CAR-T cells and PD-L1 CAR-T cells individually inhibited tumor growth in xenografts (Figures 6B and 6C). Surprisingly, Combo CAR-T cells showed a significant synergistic anti-tumor effect in xenografts, whereas bispecific CAR-T cells failed to reduce the tumor burden, and the effect was worse than that of monospecific CAR-T cells (Figures 6B and 6C). We sacrificed mice by the end of week 4 after treatment. We isolated tumors from one mouse (mouse 1) of the Combo group and one mouse (mouse 2) from the bispecific group to visualize tumor size. As shown in Figure 6D, the tumor size of a mouse (mouse 1) treated with combination CAR-T was much smaller than that of a mouse (mouse 2) treated with bispecific CAR-T cells.

To identify factors contributing to the high efficiency of the CAR-T cell combination strategy, we detected the absolute number, immunophenotype, and exhaustion of CAR-T cells isolated from mouse blood at week 2 of infusion. We found that mice receiving PD-L1 CAR, Combo CAR, or bispecific CAR had much higher CAR-T cell counts in the blood than those that received CD19 or GPC3 CAR-T cells (Figure 6E). On the other hand, the absolute number of PD-L1 CAR-T cells was higher than that of combination followed by bispecific CAR-T cells, which indicated that bispecific CAR-T cells might lose PD-L1-specific proliferation. We found that recovered CAR-T cells from the Combo mouse spleen (mouse 143) showed a higher binding ability to PD-L1 than CAR-T cells of the bispecific mouse (mouse 107) (21.2% versus 16.4%), although both CAR-T groups showed similar binding percentages in cell culture (3.28% versus 2.62%) (Figure 6F). Moreover, CAR-T cells recovered from the mouse spleen (mouse 145) showed a higher binding ability than *in-vitro-*cultured CAR-T cells, especially on PD-L1 CAR-T cells (5.93% versus 35.4%). Besides the functional capacity of endogenous T cells, the frequency of the memory T cell subset is also associated with tumor response. Here we analyzed the T differentiation subsets consisting of stem cell-like memory T cells (T_SCM_ cells; CD62L^+^CD45RA^+^CD95^+^), central memory T cells (T_CM_ cells; CD62L^+^CD45RA^−^CD95^+^), effector memory T cells (T_EM_ cells; CD62L^−^CD45RA^−^CD95^+^), and terminally differentiated effector memory T cells (T_EMRA_ cells; CD62L^−^CD45RA^+^CD95^+^) in CD4^+^CAR^+^ and CD8^+^CAR^+^ subpopulations in mouse blood after 2 weeks of infusion. As shown in Figure 6G, the combination group exhibited a significantly higher percentage of T_SCM_ cells than PD-L1 CAR-T cells and a higher frequency of T_EM_ and T_CM_ cells than GPC3 CAR-T cells in CD4^+^ and CD8^+^ subpopulations. We analyzed the expression of co-inhibitory receptors in CAR-T cells, including PD-1, LAG-3, and TIM-3. The expression level of PD-1 and LAG-3 in PD-L1 CAR-T cells was dramatically higher than GPC3 CAR-T cells in CD4^+^ and CD8^+^ subpopulations (Figure 6H).

Antigen loss is known to limit the efficacy of CAR-T cells. We evaluated GPC3 and PD-L1 expression in Hep3B tumors harvested from mice after CAR-T cell treatment by western blot. Cultured Hep3B GFP cells and IFN-γ-incubated Hep3B cells (no GFP) were controls. As shown in Figure 6I, Hep3B and Hep3B IFN-γ cells expressed GPC3, whereas only Hep3B IFN-γ cells showed high expression of PD-L1, which further validated that IFN-γ could induce PD-L1 expression in Hep3B cells. We normalized antigen expression by tumor-specific GFP. In comparison with the CD19 control, no GPC3 loss was found in the tumor treated with GPC3 CAR. The inducible expression of PD-L1 in tumors treated with PD-L1 CAR or bispecific CAR was higher than that in CD19 tumors, consistent with what we found in the cell co-culture system. Higher expression levels of both antigens, GPC3 and PD-L1, were found in the tumor treated with Combo compared with bispecific treatment. These results suggest that a combination of GPC3 CAR-T cells and PD-L1 CAR-T cells, but not bispecific CAR, synergistically killed Hep3B tumors.

## Discussion

The checkpoint molecule PD-L1 is highly expressed on many tumors in a constitutive or IFN-γ-inducible manner. IFN-γ is the critical functional cytokine released from effector T cells; however, the increased expression of PD-L1 in tumor cells binding to PD-1 in effector T cells results in T cell exhaustion and inhibition of T cell functions.^30^ We hypothesized that development of CAR-T cells targeting PD-L1 could kill solid tumors by recognizing constitutive or inducible expression of PD-L1 in the immunosuppressive TME. To test our hypothesis, we isolated a panel of anti-PD-L1 single-domain antibodies from a newly established, semi-synthetic nurse shark V_NAR_ library. The best candidate, V_NAR_ B2, showed a specific binding ability to PD-L1 and was cross-reactive with human and mouse antigens. Significantly, B2 functionally blocked the interaction between PD-L1 and PD-1. We found that the single-domain-based CAR fragment could be highly expressed in human PBMCs, indicating that single-domain antibodies are suitable to be engineered into the CAR format because they are smaller, easily expressible, and more stable.

Although several shark V_NAR_s showed a promising effect in academic and pre-clinical disease therapy research,^31^, ^32^, ^33^ the potential immunogenicity of V_NAR_s might limit their clinical development, including CAR-T cell therapy. Current FDA-approved CD19-targeted CAR-T cell therapies use murine-derived scFv (FMC63) and develop humoral and/or cellular immune responses against scFv in some individuals as one of the tumor relapse mechanisms.^34^ Kovalenko et al.^35^ humanized the framework of V_NAR_ by grafting antigen-binding domains to a human framework and observed that it largely retained antigen-binding specificity and affinity compared with the parental V_NAR_. To address this issue, we would next humanize the B2 V_NAR_ and evaluate its immunogenicity in the CAR-T format. To test the efficacy of CAR (B2) T cells *in vivo*, we established an orthotopic xenograft mouse model bearing human breast cancer. We observed significantly effective tumor inhibition and metastasis prevention caused by CAR (B2) T cells, but there was still a modest tumor recurrence at week 5 after treatment. We did not find antigen (PD-L1) loss in tumor cells, and we thought the tumor recurrence was because (1) five million CAR-T cells might not be enough to cause tumor-free, (2) the long-persistent CAR-T cells were partially exhausted because of increased expression of PD-1 (Figure 6E).

PD-L1 is not only overexpressed in a more significant number of malignancies but also in immune cells in the TME.^13^ T cells express low levels of endogenous PD-L1, which leads to development of CAR-T cells that target PD-L1 is somewhat intricate by killing PD-L1-expressing tumor cells and blocking the PD-1/PD-L1 checkpoint axis.^36^^,^^37^ To address the potential toxicity of anti-PD-L1 CAR-T cells, a previous study tested CAR-T cells targeting mPD-L1 in a syngeneic mouse colon adenocarcinoma model using immunocompetent C57BL/6 mice.^17^ These CAR-T cells increased mouse survival without apparent side effects. Antigen exposure of CAR-T cells may lead to T cell fratricide and exhaustion, impairing the proliferation and persistence of CAR-T cells *in vitro* and *in vivo*. Xie et al.^17^ reported that camelid V_H_H-based anti-mPD-L1 CAR-T cells can “self-activate” *in vitro*, and PD-L1 deficient CAR-T cells can live longer than WT CAR-T cells. However, during 7–12 days of *in vitro* co-culture with CD3/CD28 microbeads, we did not find upregulation of PD-L1 (Figure S3) or exhaustion markers (PD-1, TIM-3, and LAG-3) in activated CAR (B2) T cells compared with mock T cells. These events were probably due to PD-L1 antigen endocytosis caused by anti-PD-L1 CAR-T cells themselves.^37^^,^^38^ We did not observe any cytolytic phenomena or upregulated IFN-γ expression in cultured CAR (B2) T cells, indicating that the cytotoxicity of CAR (B2) T cells was not triggered by the T cells’ endogenous PD-L1. We consider that less tonic signaling of our shark V_NAR_-based CAR (B2) T cells may be due to the relatively low binding affinity of B2 V_NAR_. Ghorashian et al.^39^ reported enhanced proliferation and anti-tumor activity in a lower-affinity CD19 CAR compared with that in clinical high-affinity CAR (CD19) T cells, indicating that the increased immunoreceptor affinity may adversely affect T cell responses.

The combination strategy might be more feasible in solid tumor therapy to overcome tumor escape mechanisms and enhance the anti-tumor effect of CAR-T cells, such as combining CAR-T cells with monoclonal antibodies, small molecules, or bispecific CAR-T cells targeting different tumor-specific antigens.^40^^,^^41^ In our study, we found that CAR (B2) T cells could kill liver cancer cells by targeting inducible PD-L1 in the immunosuppressive TME (Figure 5D), whereas B2 V_NAR_ did not show a significant benefit in improving the cytotoxicity of CAR (GPC3) T cells even though it functionally blocked the interaction of PD-1 with PD-L1 (Figure S4). Thus, we constructed a bispecific CAR-T cell targeting the HCC tumor-specific antigen GPC3 and the inducible tumor-immunosuppressive antigen PD-L1. Surprisingly, bispecific CAR-T cells worked best *in vitro* but only slightly inhibited liver tumor progression *in vivo* and performed even worse than individual GPC3 CAR-T cells and PD-L1 CAR-T cells.

We may optimize the bispecific CAR construct in future work by engineering two CAR fragments into one construct.^19^ Combination of GPC3 CAR-T cells and PD-L1 CAR-T cells achieved a synergistic anti-tumor effect *in vivo*. A previous study reported that combination of anti-mesothelin CAR-T cells with PD-L1 CAR-T cells did not repress tumor growth synergistically in patient-derived xenograft (PDX) because PD-L1 CAR-T killed mesothelin CAR-T cells by targeting its endogenous PD-L1 antigen.^37^ We did not observe upregulated PD-L1 expression in GPC3 CAR-T cells, probably because of different CAR constructs. On the other hand, we found that expansion of the CAR-T cell count in mouse blood is highly correlated with the PD-L1 CAR construct, and this may be due to cross-recognition of B2 V_NAR_ with endogenous mouse antigen. However, CAR-T cell treatment mice were healthy and did not experience body weight loss, indicating that our PD-L1 CAR-T cells are safe in mice. Although PD-1 was highly expressed in blood-recovered CAR (B2) T cells (Figure 6G), the CAR (B2) T cells recovered from mouse spleens still efficiently lysed MDA-MB-231 tumor cells (Figure 4G), probably because of B2 V_NAR_ blocking the interaction of PD-1 with PD-L1, although not entirely.

Our results demonstrate the feasibility and efficacy of CAR-T cells targeting the tumor immunosuppressive microenvironment antigen PD-L1 against aggressive solid tumors. To improve treatment of solid tumors, future efforts should be directed at utilizing genome editing to develop “off-the-shelf,” fratricide-resistant, PD-L1-targeted CAR-T cells lacking endogenous PD-L1 and the T cell receptor α chain.

## Materials and methods

### Construction of a synthetic 18-aa CDR3 nurse shark V_NAR_ phage library

We constructed the new synthetic 18-aa CDR3 nurse shark V_NAR_ phage library based on our previous naive nurse shark library.^26^ For the V_NAR_s DNA cassettes, a non-canonical cysteine in CDR1 was mutated to tyrosine (C29Y) using the naive shark library V_NAR_ pComb3x plasmid as the template. Subsequently, a pair of randomized 18-aa CDR3 primers was designed to amplify the CDR3 loop using the PCR method. PCR products were circularized by intra-molecular self-ligation in 1 mL of ligation buffer using T4 DNA ligase (New England Biolabs, Ipswich, MA). Finally, the ligation products were purified by removing the enzymes and transformed into 500 μL of electroporation-competent TG1 cells (Lucigen, Middleton, WI) to make the phage library.

### Phage panning

The phage panning protocol has been described previously.^26^^,^^42^ The mPD-L1 protein bought from R&D Systems was used for four rounds of panning. Details are provided in the supplemental information.

### Affinity binding and blocking activity

The binding kinetics of the V_NAR_-hFc (produced by GenScript) to hPD-L1-His protein (SinoBiological) was determined using the Octet RED96 system (FortéBio) at the Biophysics Core (National Heart, Lung, and Blood Institute [NHLBI]) as described previously.^43^ The blocking activity of B2-hFc was determined using the BLI Octet platform as described previously^44^ and sandwich ELISA. We provide details in the supplemental information.

### Generation of anti-PD-L1 V_NAR_-based CAR-T cells

We generated the PD-L1-target, shark V_NAR_-based, CAR-T cell lentiviral vector following the design principle of a CAR construct published in our previous study.^10^ Briefly, the V_NAR_ fragment of B2 was subcloned into a CAR construct (pMH330). The CAR-expressing lentivirus was produced as described previously.^10^ Whole blood was collected from healthy donors under the Oklahoma Blood Institute Institutional Review Board approval. Human peripheral blood mononuclear cells (PBMCs) isolated from healthy donors were stimulated for 24 h using anti-CD3/anti-CD28 antibody-coated beads (Invitrogen) at a bead:cell ratio of 2:1 according to the manufacturer’s instructions in the presence of IL-2.

### *In vitro* cytolysis of CAR-T cells and activation assays

The cytotoxicity of CAR-T cells was determined by a luciferase-based assay. In brief, luciferase-expressing MDA-MB-231 and Hep3B tumor cells were used to establish a cytolytic assay. Cytolysis of PD-L1-targeted CAR (B2) T cells was detected by co-culture with MDA-MB-231 GL and Hep3B GL at various E/T ratios for 24 or 96 h, followed by measurement of luciferase activity using a luciferase assay system (Promega) on Victor (PerkinElmer). Supernatants were collected for TNF-α, IL-2, and IFN-γ detection using an ELISA kit (BD Biosciences). In the killing blocking assay of CAR-T cells, varying concentrations of soluble B2 protein were added to tumor CAR-T cells incubated for 24 and 48 h.

### Animal studies

5-week-old female NSG mice (NCI, Frederick, MD) were housed and treated under a protocol (LMB-059) approved by the Institutional Animal Care and Use Committee of the NIH. A total of three million MDA-MB-231 GL cells were suspended in a mixture of PBS:Matrigel (BD Biosciences) at 1:1 and inoculated into the inguinal mammary fat pad to establish the orthotopic MDA-MB-231 model. The peritoneal Hep3B xenograft tumor model was established as described previously.^10^ Tumor volume was calculated as ½ (length × width^2^) and bioluminescence intensity (Xenogen IVIS Lumina). When the average tumor size reached the indicated size, five million CAR-T cells were injected i.v. into mouse models. *Ex vivo* T cells were isolated from mouse spleens using a Miltenyi Biotec tumor dissociation kit and cultured *in vitro* with 40 ng/μL IL-2, IL-7, and IL-21 in the culture medium.

### Statistical analysis

All experiments were repeated at least three times to ensure reproducibility of results. All statistical analyses were performed using GraphPad Prism and are presented as mean ± SEM. Results were analyzed using 2-tailed unpaired Student’s t-test. A p value of less than 0.05 was considered statistically significant.

## Supplemental information

Document S1. Supplementary materials and Figures S1–S4

Document S2. Article-plus supplemental information
