# Supplementary material for: A novel PD-L1-targeted shark VNAR single-domain-based CAR-T cell strategy for treating breast cancer and liver cancer
Source: Mol Ther Oncolytics. 2022 Feb 20;24:849–63. doi: 10.1016/j.omto.2022.02.015 (PMC8917269; doi:10.1016/j.omto.2022.02.015)
Supplement: Document S2. Article-plus supplemental information [file mmc2.pdf]

Molecular Therapy
Oncolytics
Original Article

A novel PD-L1-targeted shark V_{NAR} single-domain-based CAR-T cell strategy for treating breast cancer and liver cancer

Dan Li,[1] Hejiao English,[1] Jessica Hong,[1] Tianyuzhou Liang,[1] Glenn Merlino,[2] Chi-Ping Day,[2] and Mitchell Ho[1]

[1]Laboratory of Molecular Biology, Center for Cancer Research, National Cancer Institute, Bethesda, MD 20892, USA; [2]Laboratory of Cancer Biology and Genetics, Center for Cancer Research, National Cancer Institute, Bethesda, MD 20892, USA

Chimeric antigen receptor (CAR)-T cell therapy shows excellent potency against hematological malignancies, but it remains challenging to treat solid tumors, mainly because of a lack of appropriate antigenic targets and an immunosuppressive tumor microenvironment (TME). The checkpoint molecule programmed death-ligand 1 (PD-L1) is widely overexpressed in multiple tumor types, and the programmed death-ligand 1 (PD-1)/PD-L1 interaction is a crucial mediator of immunosuppression in the TME. Here we constructed a semi-synthetic shark V_{NAR} phage library and isolated anti-PD-L1 single-domain antibodies. Among these V_{NAR}s, B2 showed cross-reactivity to human, mouse, and canine PD-L1, and it partially blocked the interaction of human PD-1 with PD-L1. CAR (B2) T cells specifically lysed human breast cancer and liver cancer cells by targeting constitutive and inducible expression of PD-L1 and hindered tumor metastasis. Combination of PD-L1 CAR (B2) T cells with CAR T cells targeted by GPC3 (a liver cancer-specific antigen) regresses liver tumors in mice. We concluded that PD-L1-targeted shark V_{NAR} single-domain-based CAR-T therapy is a novel strategy to treat breast and liver cancer. This study provides a rationale for potential use of PD-L1 CAR-T cells as a monotherapy or in combination with a tumor-specific therapy in clinical studies.

INTRODUCTION

Adoptive cell therapy (ACT), particularly chimeric antigen receptor (CAR)-T cell therapy, has shown great potency as one of the most effective cancer immunotherapies.[1–3] CARs are synthetic receptors consisting of an extracellular domain, a hinge region, a transmembrane domain, and intracellular signal domains (e.g., CD3-zeta, CD28, and 41BB) that initiate T cell activation.[4–6] CARs can promote non-major histocompatibility complex (MHC)-restricted recognition of cell surface components, bind tumor antigens directly, and trigger a T cell anti-tumor response.[7] So far, CAR-T cells targeting the B cell antigen CD19 have shown clinical success in individuals with advanced B cell lymphoma, which led to their approval by the US Food and Drug Administration (FDA).[3,8] However, translation of CAR-T therapy to solid tumors is more difficult because of a lack of appropriate antigenic targets and the complex immunosuppressive tumor microenvironment (TME). Recently, the proteins glypican-2 (GPC2),[9] glypican-3 (GPC3),[10] and mesothelin[11,12] have been reported as emerging antigens for CAR-T therapy for treatment of solid tumors and development for clinical trials. However, not all tumors express particular surface antigens suitable for CAR recognition. Tumor heterogeneity makes targeted therapy more challenging. Programmed death-ligand 1 (PD-L1 or CD274) has aberrantly high expression on multiple tumor types through oncogenic signaling[13] and is induced by pro-inflammatory factors such as interferon (IFN)-γ in the immunoreactive TME.[14] It has been shown that PD-L1 expressed on tumors can induce T cell tolerance and avoid immune destruction through binding with its ligand programmed cell death protein 1 (PD-1) on T cells, which may be one of the main reasons for the poor effect of CAR-T cells in solid tumors.[15] Clinically, antibody-based PD-1/PD-L1 antagonists have been reported to induce durable tumor inhibition, especially in melanoma, non-small cell lung cancer, and renal cancer. However, the response rate remains poor in other types of advanced solid tumors.[16] PD-L1-targeting camelid V_HH-nanobody-based CAR-T cells have been shown to delay tumor growth in a syngeneic mouse melanoma model.[17]

PD-L1-targeting CAR natural killer (NK) cells inhibit growth of triple-negative breast cancer (TNBC), lung cancer, and bladder tumors engrafted in non-obese diabetic (NOD) severe combined immunodeficiency (SCID) gamma (NSG) mice.[18] Bispecific Trop2/PD-L1 CAR-T cells targeting Trop2 and PD-L1 demonstrate the improved killing effect of CAR-T cells in gastric cancer.[19] PD-L1-targeted CAR-T cell therapy is presumed to kill PD-L1-overexpressing tumor cells and block the PD-1/PD-L1 immune checkpoint, significantly enhancing anti-tumor activity in solid tumors.

Single-chain variable fragment (scFv), a type of recombinant antibody, commonly serves as the antigen recognition region of a CAR construct. It consists of variable heavy (V_H) and variable light (V_L)

Received 4 August 2021; accepted 15 February 2022;
https://doi.org/10.1016/j.omto.2022.02.015.

Correspondence: Mitchell Ho, Laboratory of Molecular Biology, National Cancer Institute, National Institutes of Health, 37 Convent Drive, Room 5002, Bethesda, MD 20892-4264, USA.
E-mail: homi@mail.nih.gov

This is an open access article under the CC BY license (http://creativecommons.org/licenses/by/4.0/).

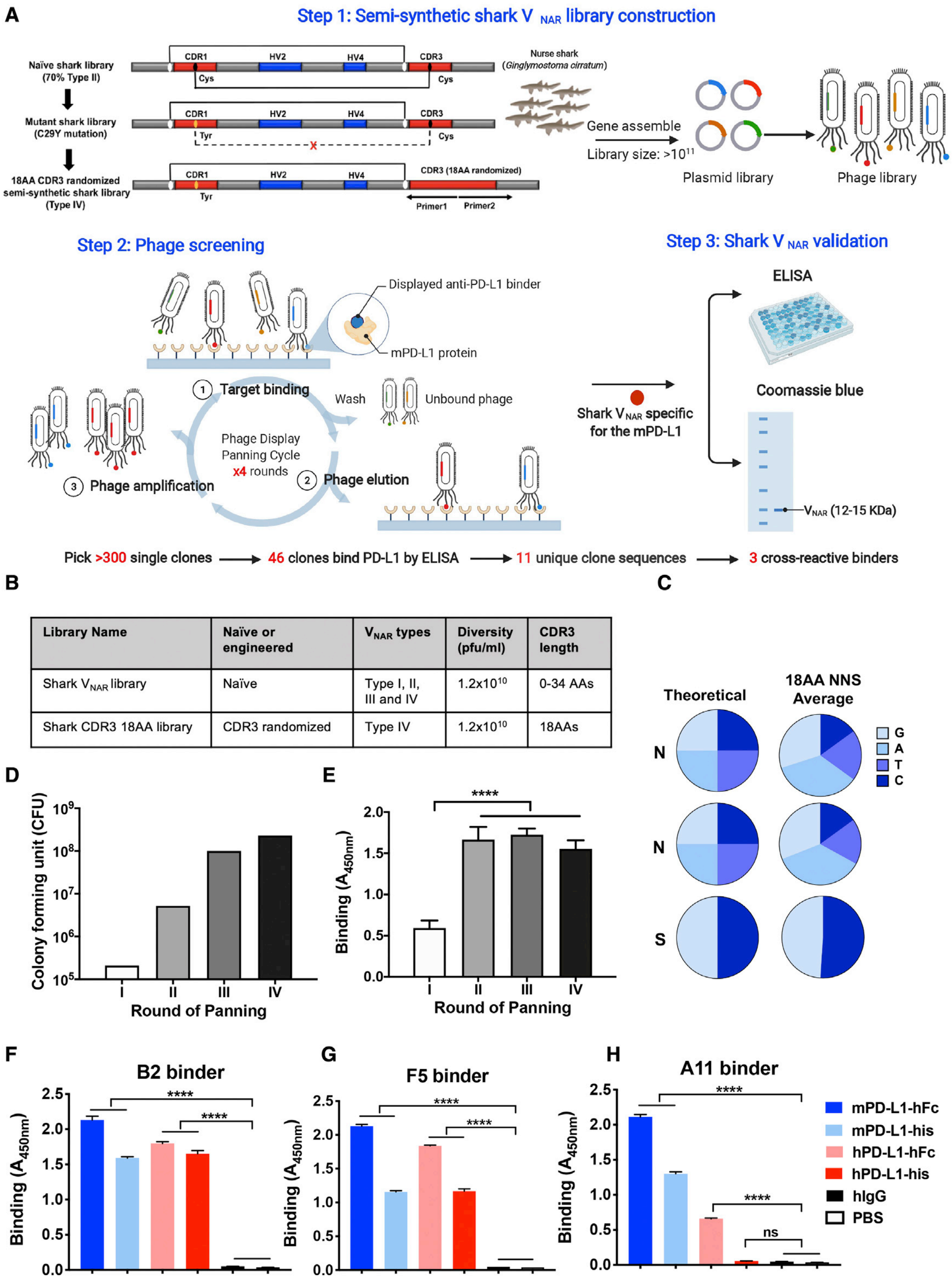

Library Name	Naïve or engineered	V_{NAR} types	Diversity (pfu/ml)	CDR3 length
Shark V_{NAR} library	Naïve	Type I, II, III and IV	$1.2x10^{10}$	0-34 AAs
Shark CDR3 18AA library	CDR3 randomized	Type IV	$1.2x10^{10}$	18AAs

Figure 1. Isolation of the anti-PD-L1 single-domain antibody by phage display from an engineered semi-synthetic shark V_{NAR} phage library

(A) Circuit of three steps of library construction and phage panning. An 18-aa randomized CDR3 semi-synthetic shark V_{NAR} phage library was constructed by PCR mutation and gene assembly. After 3–5 rounds of phage panning, anti-mPD-L1 V_{NAR}s were isolated from the phage library and validated by phage ELISA and protein purification

(legend continued on next page)

chains connected by a flexible linker ($Gly_4Ser)_3$. However, folding of an artificially engineered scFv can affect the specificity and affinity of the CAR for its target antigen.[20] Alternatively, the antigen-binding domain of naturally occurring single-domain antibodies (heavy chain only) from camelids (V_HH)[21] and sharks (V_{NAR})[22] have beneficial properties for engineering of CARs. They are small (12–15 kDa), easy to express, and capable of binding concave and hidden epitopes inaccessible to conventional antibodies.[23] Remarkably, shark V_{NAR}s have unique features distinct from camel V_HHs—they have great diversity and are evolutionarily derived from an ancient single domain that functions as a variable domain in B cell and T cell receptors.[24,25] We previously constructed a V_{NAR} phage display library from six nurse sharks.[26] Currently, several shark V_{NAR}s are in pre-clinical research, and their therapeutic and biotechnological applications are under intensive investigation.[27–29] In this study, to improve the diversity of the shark V_{NAR} repertoire, we constructed a semi-synthetic shark V_{NAR} phage library with randomized third complementarity-determining regions (CDR3s) 18 amino acids (aa) in length. Of the three cross-reactive binders, only V_{NAR} B2 could functionally block the interaction between human PD-L1 and PD-1. More importantly, B2-based CAR-T cells successfully inhibited tumor growth in xenograft mouse models of TNBC and hepatocellular carcinoma (HCC). Interestingly, the combination of anti-PD-L1 CAR (B2)-T cells and anti-GPC3 CAR-T cells synergistically demonstrated better efficacy than single-antigen-targeted CAR-T cells in the HCC xenograft mouse model, highlighting the feasibility and efficacy of shark V_{NAR}-based CAR-T cells targeting PD-L1 in solid tumors.

RESULTS

Construction of a semi-synthetic shark V_{NAR} single-domain library

We previously constructed a naive shark V_{NAR} library from 6 naive adult nurse sharks (*Ginglymostoma cirratum*) with a size of 1.2×10^{10} plaque-forming units (PFUs)/mL.[25,26] To improve the diversity and utility of the shark V_{NAR} library, here we developed a semi-synthetic randomized CDR3 shark V_{NAR} library (referred to as the ’18-aa CDR3 shark library). As illustrated in Figure 1A, 70% of V_{NAR}s in the naive shark library are type II, containing two canonical cysteines located at aa 21 and 82 to form a disulfide bond and at least one extra cysteine in CDR1 and CDR3 to form an interloop disulfide bond. Because the type IV V_{NAR} sequence is the closest to its mammalian counterpart, such as human V_H with only a pair of canonical cysteines (one before CDR1 and the other before CDR3), we mutated C29Y in CDR1 and randomized the CDR3 loop region to change all four types (type I, II, III, and IV) to type IV V_{NAR}s. The diversity of the new semi-synthetic library is approximately 1.2×10^{10} PFUs/mL, which is comparable with our original naive shark V_{NAR} library (Figures 1A and 1B). To assess the randomness of sequence modification, we estimated the average nucleotide ratio at each CDR3 residue based on sequencing analysis and found that the CDR3 nucleotides were completely randomized with the desired ATGC bases ratios (Figure 1C).

Isolation of cross-species V_{NAR} single domains with high affinity for PD-L1

To identify the anti-PD-L1 shark V_{NAR} that functions in the murine tumor environment, we used mouse PD-L1 (mPD-L1) protein as an antigen to screen the new semi-synthetic shark library (Figure 1A). After four rounds of panning, ≈1,000-fold enrichment of eluted phage colonies was obtained (Figure 1D). We also observed enhanced binding to PD-L1 after the first round of phage panning (Figure 1E). At the end of the fourth round of panning, 46 individual clones were identified to bind mPD-L1 protein by the monoclonal phage enzyme-linked immunosorbent assay (ELISA), and 11 unique binders were confirmed by subsequent sequencing. Three PD-L1-specific V_{NAR}s (B2, A11, and F5) finally showed cross-reactivity to mPD-L1 and human PD-L1 (hPD-L1) protein in the His tag or hFc tag format, as shown by monoclonal phage ELISA (Figures 1F–1H).

To determine the antigen specificity of shark V_{NAR}s, we established PD-L1 knockout (KO) single clones by CRISPR-Cas9 technology in a human TNBC cell line, MDA-MB-231. To enhance the PD-L1 KO efficiency, we designed two single guide RNAs (sgRNAs) targeting the promoter of the endogenous PD-L1 gene (Figure 2A). All three individual cell clones were confirmed by loss of PD-L1 expression (Figure 2A), and clone 1 was used further in the present study. To determine the cross-species reactivity of anti-PD-L1 shark V_{NAR}s against native PD-L1, three PD-L1-positive tumor cell lines, including a human breast cancer cell line, a mouse melanoma cell line, and a canine melanoma cell line, were used to evaluate the binding ability of B2, A11, and F5. As shown in Figure 2B, B2 and F5 bind human antigens and cross-react with mouse and canine antigens. B2 showed a higher binding ability to human and mouse antigens than F5. A11 binds canine antigen but not human or mouse antigen. In contrast, no binding was shown on PD-L1 KO cells, indicating that the binding ability of shark V_{NAR}s is antigen specific.

We further produced V_{NAR}-hFc fusion proteins and incubated them with hPD-L1-His protein on the biolayer interferometry (BLI) Octet platform to determine binding kinetics. The K_D value of B2 was 1.7 nM and 1.4 nM at a concentration of 100 nM and 50 nM, respectively (Figure 2C), whereas F5 failed to yield an accurate K_D value because it showed slight non-specific binding to the nickel-charged tris-nitrilotriacetic acid (Ni-NTA) sensor on Octet. To examine whether B2 could functionally block the interaction between human PD-1 (hPD-1) and hPD-L1, we developed a blocking assay based on

technologies. (B) Information regarding the new shark V_{NAR} library compared with the pre-synthetic V_{NAR} library. (C) Pie chart of the percentages of average nucleotide (ACTG) ratio at each randomization NNS (where N = A/C/G/T, and S = C/G). (D) Phage-displayed single-domain antibody clones were identified against recombinant mPD-L1-His after four rounds of panning. A gradual increase in phage titers was observed during each round of panning. (E) Polyclonal phage ELISA from the output phage of each round of panning. (F–H) Cross-reactivity of anti-PD-L1 B2 (F), A11 (G), and F5 (H) to mPD-L1 and hPD-L1 protein within the His tag or hFc tag by monoclonal phage ELISA.

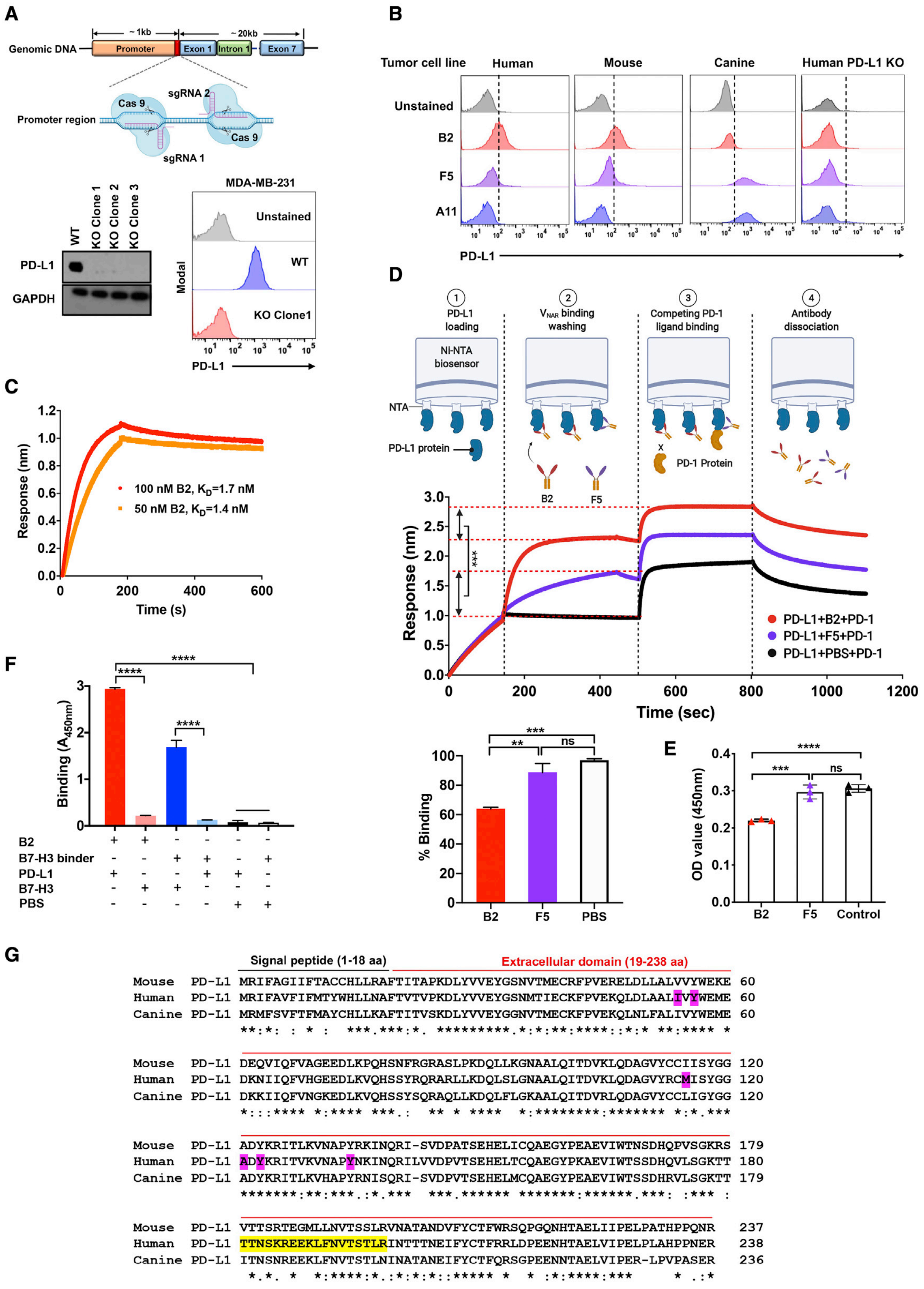

(legend on next page)

BLI technology. As shown in Figure 2D, B2 partially blocked the interaction of hPD-1 with hPD-L1 compared with the PBS control. In contrast, F5 showed positive binding to hPD-L1 but could not block the hPD-1/hPD-L1 interaction. We conducted sandwich ELISA to validate the functional blocking capacity of B2. It showed that the V_{NAR} did partially block the interaction of hPD-1 with hPD-L1 (Figure 2E). In addition, V_{NAR} B2 specifically binds to hPD-L1 but not human B7-H3, another B7-CD28 family member (Figure 2F).

To identify the binding epitope of anti-PD-L1 nanobodies, we synthesized a peptides array based on the hPD-L1 extracellular domain (ECD) consisting of a total of 24 peptides. As shown in Figures S1 and 2G, F5 and B2 strongly bind to peptide 19 (TTNSKREEKLFNVTSTLR), whereas A11 did not bind to any peptides.

We successfully identified functionally cross-species anti-PD-L1 shark V_{NAR} with high affinity.

Anti-PD-L1 CAR (B2) T cells kill breast cancer cells

Based on flow cytometry analysis, we found that PD-L1 was highly expressed in multiple human tumor types, including breast cancer (MDA-MB-231), ovarian cancer (IGROV-1, OVCAR8, and NCI-ADR-RES), pancreatic cancer (KLM1 and SU8686), and lung cancer (EKVX), suggesting that PD-L1 is a putative pan-cancer antigen (Figure 3A). To determine the application of our shark V_{NAR}s to a CAR-T cell therapeutic approach, we constructed CARs containing the B2 V_{NAR} fragment as the antigen recognition region, along with 4-1BB, CD3ζ signaling domains, and a truncated human epidermal growth factor receptor (hEGFRt) cassette to gauge transduction efficiency and to switch off CAR (Figure 3B). The transduction efficiency of CAR (B2) T was high (~90%) (Figure 3C). During days 7–12, non-transduced mock T and CAR (B2) T cells showed indistinguishable expression of exhaustion markers (PD-1 and TIM-3) compared with each other, whereas a slightly higher expression of LAG-3 was found in CAR (B2) T cells compared with mock T cells (Figure 3D). MDA-MB-231 is a highly aggressive, invasive, and poorly differentiated TNBC cell line with limited treatment options. We therefore engineered it to overexpress GFP/luciferase (GL) to establish a luciferase-based cytolytic assay. Mock T and CAR (B2) T cells were incubated with MDA-MB-231 GL cells for 24 or 96 h. As shown in Figure 3E, CAR (B2) T cells effectively lysed tumor cells in a 2-fold dose-dependent manner compared with mock T cells. Moreover, the 96-h incubation procedure improved the cytotoxicity of CAR (B2) T cells even at the lowest effector:target (E/T) ratio of 1:3. To investigate whether the cytolytic activity of CAR (B2) T cells is antigen dependent, we incubated CAR (B2) T cells with MDA-MB-231 PD-L1 KO cells. This showed that CAR-T cells could not kill antigen KO cells (Figure 3E). A dramatically higher level of tumor necrosis factor alpha (TNF-α), interleukin-2 (IL-2), and IFN-γ was released from CAR T cells at E/T ratios of 5:1 and 2.5:1, whereas there was minimum cytokine production in mock T cells (Figure 3F). These results suggested that V_{NAR}-based CAR-T cells could lyse tumor cells by efficiently targeting PD-L1. We included a corresponding soluble B2 V_{NAR} in the co-culture system to detect whether it could affect the cytotoxicity of CAR (B2) T cells by competitively blocking the recognition site on tumor cells. As shown in Figure 3G, inclusion of the B2 single domain significantly inhibited the cytolytic activity of CAR (B2) T cells. In contrast, no specific lysis in tumor cells was found in coincubation with mock T cells or tumor cells alone in the presence of B2. We concluded that CAR (B2) T cells could specifically lyse PD-L1-positive human tumor cells.

Anti-PD-L1 CAR (B2) T cells inhibit orthotopic breast cancer growth in mice

To evaluate the anti-tumor efficacy of PD-L1-specific CAR-T cells in mice, we established an orthotopic breast tumor xenograft model by implanting MDA-MB-231 GL cells into the fourth mouse mammary fat pad. Seventeen days after tumor inoculation, mice were infused intravenously (i.v.) with CAR (B2) T cells or antigen-mismatched CAR (CD19) T cells (Figure 4A). We used bioluminescence intensity and tumor volume to track the anti-tumor efficacy of CAR-T cells. At weeks 3–4 after treatment, we found that the tumor bioluminescence signal was saturated, as measured by the IVIS imaging system, suggesting that bioluminescence imaging might not be a suitable method to accurately measure tumor size, in particular for large tumors. Mice were followed up to 8 weeks after CAR-T cell infusion. We euthanized one CAR (CD19) T cell-treated mouse (mouse 1) and two CAR (B2) T cell-treated mice (mice 2 and 3) after week 3 to perform CAR-T cell persistence analysis (Figure 4B). We observed that CAR (B2) T cells dramatically reduced the breast tumor burden (Figures 4B and 4C) without a marked loss of body weight (Figure 4D). Tumors metastasized in control group mice after 5 weeks of CAR (CD19) T cell infusion (Figures 4B and 4E). We did not observe tumor metastases in mouse liver or lungs with CAR (B2) T cell infusion (Figures 4B and 4E). These data indicated that CAR (B2) T cells could treat breast cancer metastatic lesions. To determine CAR-T cell persistence, we recovered CAR (CD19) and CAR (B2) T cells from mouse spleen

Figure 2. Verification of the specific binding and blocking ability of anti-PD-L1 shark V_{NAR}s

(A) Schematic of constructing the PD-L1 KO MDA-MB-231 cell line using CRISPR-Cas9. Two sgRNAs were designed to target the promoter of the endogenous PD-L1 gene. Single PD-L1 KO clones were validated by western blot and flow cytometry. (B) The cross-reactive binding of anti-PD-L1 V_{NAR}s to native PD-L1 as determined by flow cytometry. Three different tumor cell lines (the human breast cancer cell line MDA-MB-231, murine melanoma cell line B8979HC, and canine tumor cell line Jones) were stained with V_{NAR}s. (C) Binding kinetics of B2-hFc to hPD-L1 protein. (D-E) Blocking activity of V_{NAR}-hFc to the interaction of hPD-L1 and hPD-1 as determined by the Octet platform (D) and sandwich ELISA (E). (F) Specific binding of B2 to hPD-L1 and hB7-H3. (G) Epitope mapping of individual B2, F5, and A11 and sequence alignment of the PD-L1 ECD region of human, mouse, and dog. Conserved residues are marked with asterisks, residues with similar properties between variants are marked with colons, and residues with marginally similar properties are marked with periods. The main binding residues of the hPD-L1 identified previously that interact with PD-1 are shaded in magenta. The binding peptides of B2 to hPD-L1 are highlighted in yellow. Values represent mean ± SEM. $^{**}p < 0.01$; $^{***}p < 0.001$; $^{****}p < 0.0001$; ns, not significant.

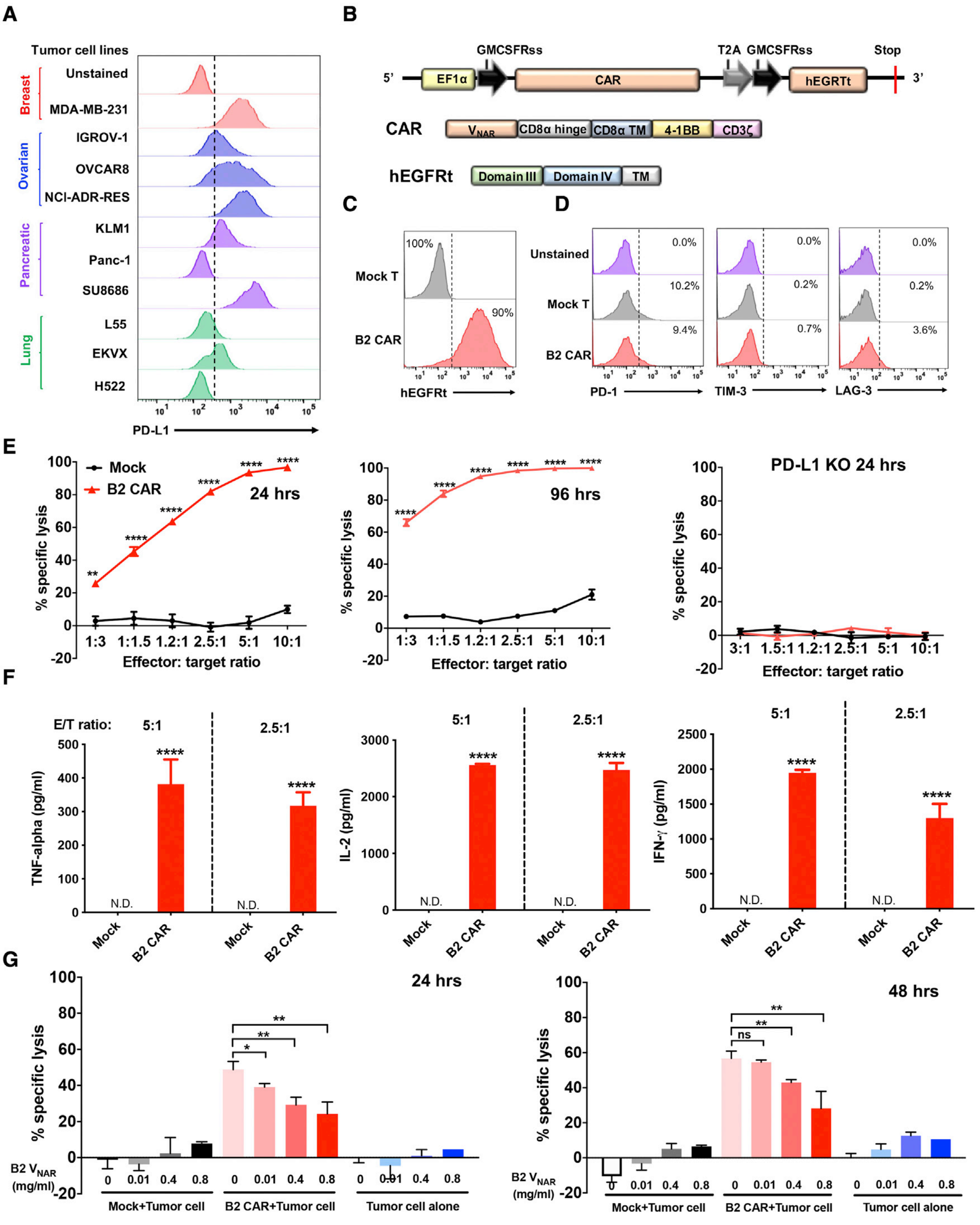

Figure 3. PD-L1 specific V_{NAR}-based CAR-T cells exhibit antigen specific cytotoxicity against MDA-MB-231

(A) Surface PD-L1 expression on multiple human tumor types as determined by flow cytometry. (B) Construct of CAR (B2) T cells, where CAR and hEGFRt are expressed separately by the self-cleaving T2A ribosomal skipping sequence. (C) The transduction efficiency of CAR (B2) in T cells was determined by hEGFRt expression. Non-transduced T cells were the mock control. (D) Exhaustion marker expression on *in-vitro*-cultured mock T cell and CAR (B2) T cell populations. (E) Cytolytic activity of CAR (B2) T cells after 24 or 96 h of incubation with MDA-MB-231 GL or PD-L1 KO MDA-MB-231 GL, respectively, in a 2-fold dose-dependent manner. (F) TNF-α, IL-2, and IFN-γ concentrations in the supernatants of the killing assay at E/T ratios of 5:1 and 2.5:1 in (D), as measured by ELISA. (G) The monovalent B2 V_{NAR} protein specifically inhibited the cytotoxicity of CAR (B2) T cells on MDA-MB-231 cells after 24- and 48-h incubation. Statistical analyses are shown from three independent experiments. Values represent mean ± SEM. $^{**}p < 0.01$; $^{***}p < 0.001$; $^{****}p < 0.0001$.

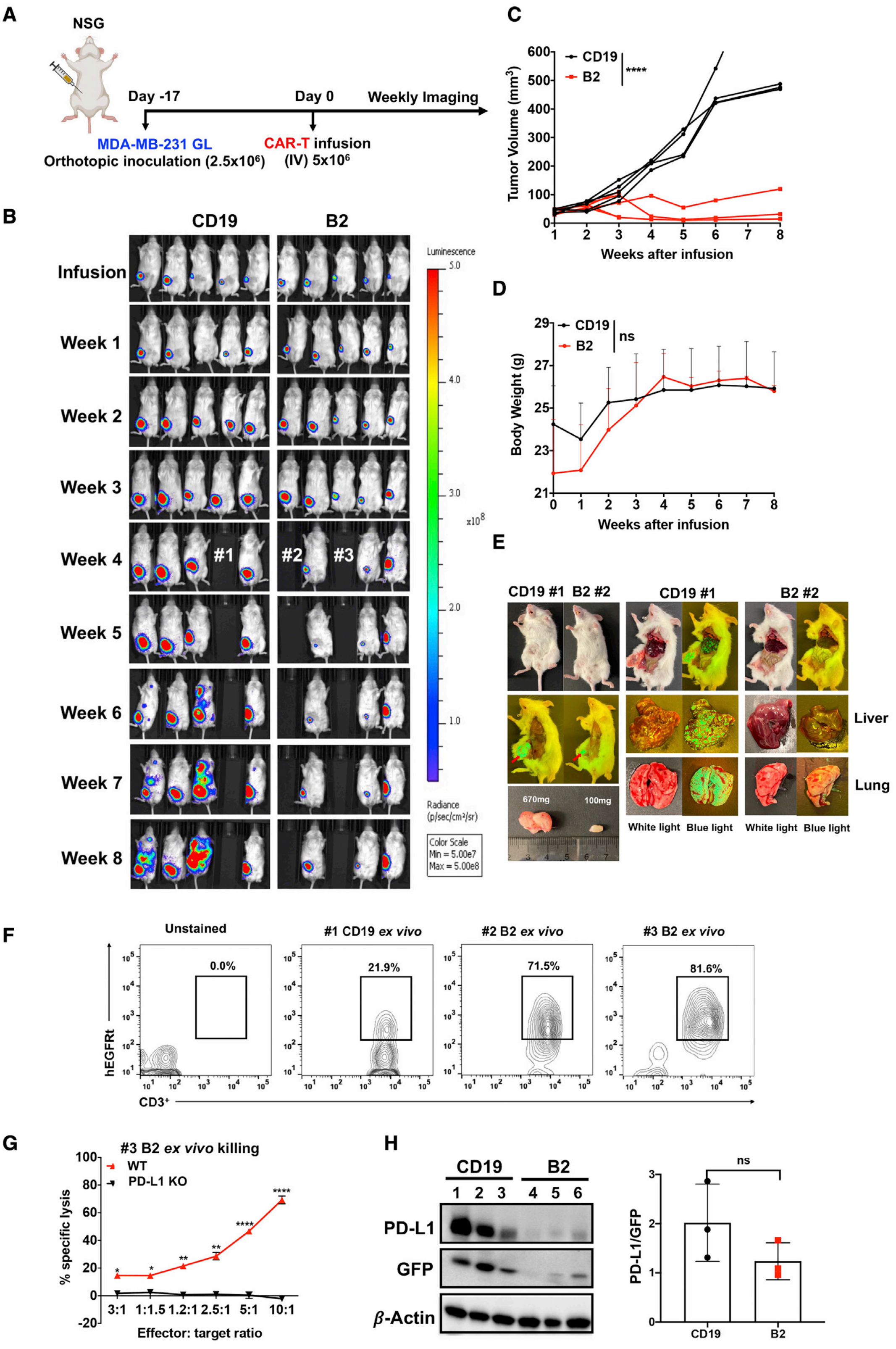

(legend on next page)

(mice 1, 2, and 3) at week 4. We found that *ex vivo* CAR (B2) T cells recovered from mice had a comparable persistence after 3 weeks of infusion (Figure 4F). These spleen-isolated CAR (B2) T cells still exhibited significant *ex vivo* cytotoxicity against PD-L1-positive tumor cells (wild type [WT]) compared with PD-L1 KO cells (Figure 4G), which suggested that these *in vivo* persistent CAR (B2) T cells remained robust. By the end of week 8, mice were euthanized, and tumors were isolated from 6 mice to analyze antigen expression after CAR-T cell treatment *in vivo*. We normalized PD-L1 expression by tumor-specific GFP expression and found that there was no significant difference in PD-L1 expression between the CAR (CD19) T cell group and CAR (B2) T cell group (Figure 4H).

CAR (B2) T cells kill liver cancer cells by targeting inducible PD-L1

We found inducible but not constitutive expression of PD-L1 in the liver cancer cell line Hep3B upon IFN-γ stimulation (Figure 5A). PD-L1 expression in Hep3B cells increased within a short period of IFN-γ incubation (4 h) and reached a peak at 8 h. Inducible PD-L1 expression decreased slowly and gradually over time after IFN-γ removal but remained for up to 96 h. These data suggested that PD-L1 expression in Hep3B cells could be up-regulated quickly and sustained for a relatively long time upon IFN-γ stimulation, suitable for PD-L1 CAR-T cell recognition and function. We also found inducible PD-L1 expression in Hep3B tumor cells co-cultured with CAR (B2) T cells for 24 h, which may in no small part be due to release of massive IFN-γ from CAR-T cells (Figure 5B). To test the anti-tumor effect of CAR (B2) T cells by targeting inducible PD-L1 *in vivo*, we established a xenograft mouse model with intraperitoneal (i.p.) injection of Hep3B GL tumor cells. After 12 days of tumor inoculation, mice were infused i.p. with CAR-T cells (Figure 5C). Four of 5 CAR (B2) T cell-treated mice showed a significant decrease in tumor growth compared with the control CAR (CD19) T cell group after 3 weeks of infusion (Figures 5D and 5E). Based on this observation, we believe that CAR (B2) T cells might benefit liver cancer therapy.

Anti-PD-L1 CAR-T cells improve the killing effect of anti-GPC3 CAR-T cells *in vitro*

Our previous study developed GPC3-targeted CAR-T cells as an emerging liver cancer therapy.[10] We observed that CAR (GPC3) T cells killed Hep3B tumor cells efficiently but inducible PD-L1 expression was found in the Hep3B cells co-cultured with CAR (GPC3) T cells rather than control CAR (CD19) T cells (Figure 5F), which may allow cancers to evade the host immune system. Therefore, we hypothesized that eliminating inducible PD-L1-positive tumor cells by CAR (B2) T cells in the TME could improve the efficacy of CAR-T cells targeting liver cancer. To test our hypothesis, we designed two strategies: bispecific CAR-T cells targeting PD-L1 and GPC3 and combination (Figure 5G). We produced bispecific CAR-T cells by co-transduction with a CAR (GPC3) and CAR (B2) lentivirus (Figure 5G). The transduction efficiency of bispecific CAR was similar to PD-L1 or GPC3 CAR based on hEGFRt expression (Figure S2A). We found that bispecific CAR had slightly weaker binding to PD-L1-hFc than PD-L1 CAR (21.9% versus 28.3%), whereas its binding ability to GPC3-hFc was similar to that of GPC3 CAR (17.3% versus 17.9%) (Figure S2B). To compare their anti-tumor effects, we incubated all seven groups of CAR-T cells and mock T cells with Hep3B cells for 24 and 72 h (Figure 5G). As shown in Figure 5H, the cytotoxicity of bispecific CAR was significantly higher than either of the monospecific CAR, especially at 72 h of incubation time. PD-L1 CAR-T cells could improve the efficiency of GPC3 CAR-T cells in a dose-dependent manner (MOI 2.5 versus 5).

The bispecific and combination CAR-T cell strategies showed dramatically higher TNF-α, IL-2, and IFN-γ levels than monospecific CAR-T cells upon tumor cell stimulation (Figure 5I). Therefore, we concluded that the bispecific CAR-T cell and combined CAR-T cell strategies significantly improved the activity of CAR-T cells in liver cancer by targeting PD-L1 and GPC3.

Combination of PD-L1 CAR-T cells and GPC3 CAR-T cells achieves a synergistic anti-tumor effect in mice

To further analyze the functions of bispecific and combination CAR-T cell strategies in response to liver cancer *in vivo*, we established a Hep3B xenograft mouse model. Mice bearing Hep3B tumors were divided into five groups and infused with five million equivalents of CD19 CAR-T cells, GPC3 CAR-T cells, PD-L1 CAR-T cells, bispecific CAR-T cells, and a combination of 2.5 million GPC3 CAR-T cells and PD-L1 CAR-T cells (referred to as “Combo”), respectively. The tumor luciferase signal was evaluated by bioluminescence imaging weekly, and T cells isolated from week 2 mouse blood were analyzed by flow cytometry (Figure 6A). Compared with CD19 CAR-T cells, monospecific therapy of GPC3 CAR-T cells and PD-L1 CAR-T cells individually inhibited tumor growth in xenografts (Figures 6B and 6C). Surprisingly, Combo CAR-T cells showed a significant synergistic anti-tumor effect in xenografts, whereas bispecific CAR-T cells failed to reduce the tumor burden, and the effect was worse than that of monospecific CAR-T cells (Figures 6B and 6C). We sacrificed mice by the end of week 4 after treatment. We isolated tumors from one mouse (mouse 1) of the Combo group and one mouse (mouse 2) from the bispecific group to visualize tumor size. As shown in Figure 6D, the tumor size of a mouse (mouse 1) treated with combination CAR-T was much smaller than that of a mouse (mouse 2) treated with bispecific CAR-T cells.

Figure 4. Tumor regression in the orthotopic MDA-MB-231 xenograft mouse model by anti-PD-L1 CAR (B2) T cell infusion

(A) Schematic of the MDA-MB-231 orthotopic xenograft NSG model infused i.v. with five million CAR (B2) T cells and CAR (CD19) T cells after 17 days of tumor inoculation. (B) Representative bioluminescence image of MDA-MB-231 tumor growth in the orthotopic model. (C) Tumor size of every mouse measured by a digital caliper [$V = 1/2(\text{length} \text{ width}^2)$]. ****$p < 0.0001$. (D) Body weight of mice. Values represent mean ± SEM. (E) Representative pictures showing the restriction of tumor metastasis in CAR (B2) T cell-infused mice. (F-G) The persentage of persistent hEGFRt$^+$ CAR-T cells in the total CD3$^+$ human T cells recovered from mice after 3 weeks of CAR-T cell infusion (F) and their *ex vivo* killing on MDA-MB-231 tumor cells (G). (H) Detection of PD-L1 expression in MDA-MB-231 tumor xenografts by western blotting.

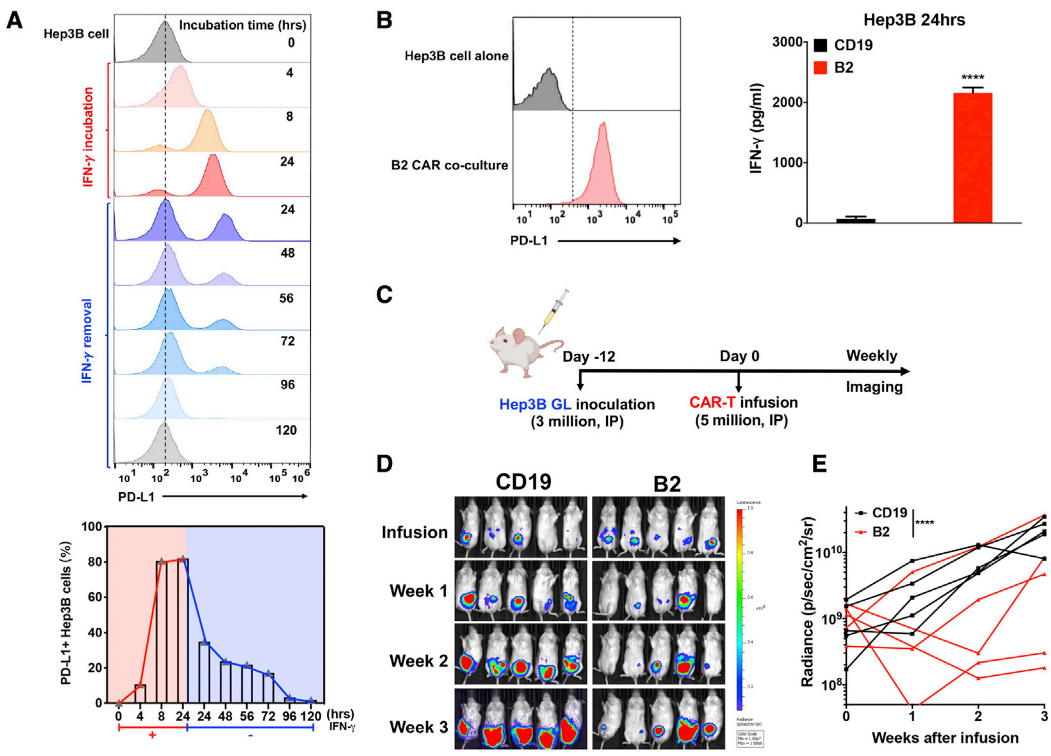

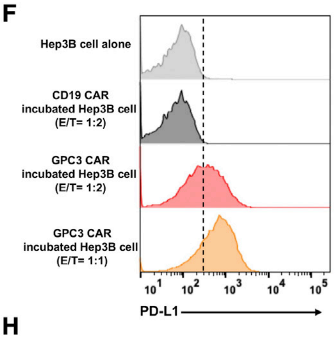

G

No.	Samples name	Information
1	Mock	Un-transduced T cells
2	GPC3	Anti-GPC3 CAR-T
3	PD-L1	Anti-PD-L1 CAR (B2) T
4	Bispecific (MOI 5)	Bispecific GPC3/PD-L1 CAR-T; B2 MOI=5
5	Bispecific (MOI 2.5)	Bispecific GPC3/PD-L1 CAR-T; B2 MOI=2.5
6	Combo-mock	Combination anti-GPC3 CAR-T with mock T
7	Combo	Combination anti-GPC3 CAR-T with anti-PD-L1 CAR (B2) T
*MOI: Multiplicity of Infection		

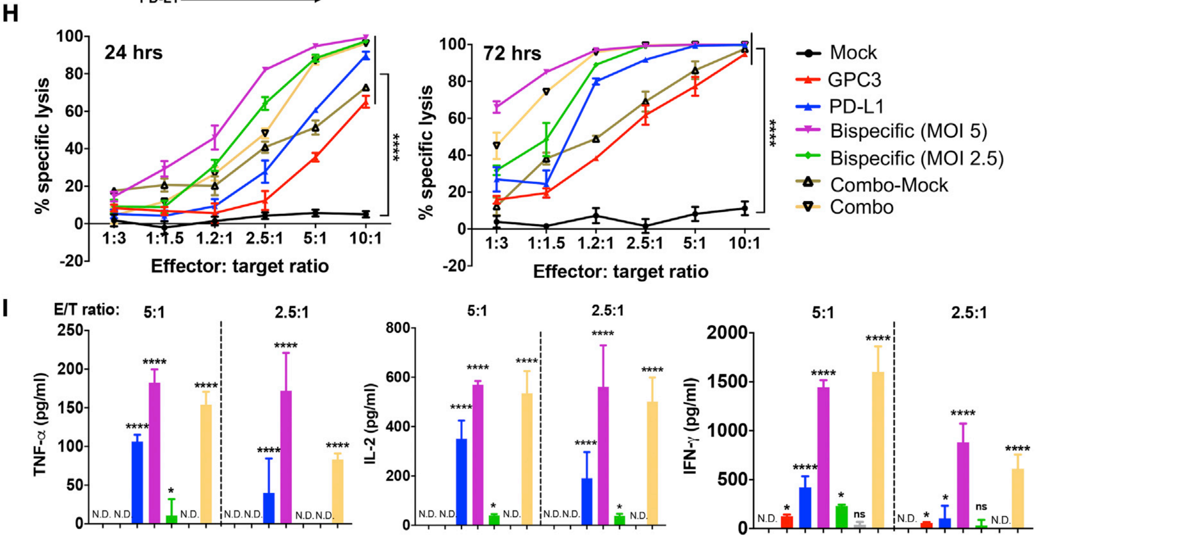

(legend on next page)

To identify factors contributing to the high efficiency of the CAR-T cell combination strategy, we detected the absolute number, immunophenotype, and exhaustion of CAR-T cells isolated from mouse blood at week 2 of infusion. We found that mice receiving PD-L1 CAR, Combo CAR, or bispecific CAR had much higher CAR-T cell counts in the blood than those that received CD19 or GPC3 CAR-T cells (Figure 6E). On the other hand, the absolute number of PD-L1 CAR-T cells was higher than that of combination followed by bispecific CAR-T cells, which indicated that bispecific CAR-T cells might lose PD-L1-specific proliferation. We found that recovered CAR-T cells from the Combo mouse spleen (mouse 143) showed a higher binding ability to PD-L1 than CAR-T cells of the bispecific mouse (mouse 107) (21.2% versus 16.4%), although both CAR-T groups showed similar binding percentages in cell culture (3.28% versus 2.62%) (Figure 6F). Moreover, CAR-T cells recovered from the mouse spleen (mouse 145) showed a higher binding ability than *in-vitro*-cultured CAR-T cells, especially on PD-L1 CAR-T cells (5.93% versus 35.4%). Besides the functional capacity of endogenous T cells, the frequency of the memory T cell subset is also associated with tumor response. Here we analyzed the T differentiation subsets consisting of stem cell-like memory T cells (T_{SCM} cells; $CD62L^+CD45RA^+CD95^+$), central memory T cells (T_{CM} cells; $CD62L^+CD45RA^-CD95^+$), effector memory T cells (T_{EM} cells; $CD62L^-CD45RA^-CD95^+$), and terminally differentiated effector memory T cells (T_{EMRA} cells; $CD62L^-CD45RA^+CD95^+$) in $CD4^+CAR^+$ and $CD8^+CAR^+$ subpopulations in mouse blood after 2 weeks of infusion. As shown in Figure 6G, the combination group exhibited a significantly higher percentage of T_{SCM} cells than PD-L1 CAR-T cells and a higher frequency of T_{EM} and T_{CM} cells than GPC3 CAR-T cells in $CD4^+$ and $CD8^+$ subpopulations. We analyzed the expression of co-inhibitory receptors in CAR-T cells, including PD-1, LAG-3, and TIM-3. The expression level of PD-1 and LAG-3 in PD-L1 CAR-T cells was dramatically higher than GPC3 CAR-T cells in $CD4^+$ and $CD8^+$ subpopulations (Figure 6H).

Antigen loss is known to limit the efficacy of CAR-T cells. We evaluated GPC3 and PD-L1 expression in Hep3B tumors harvested from mice after CAR-T cell treatment by western blot. Cultured Hep3B GFP cells and IFN-γ-incubated Hep3B cells (no GFP) were controls. As shown in Figure 6I, Hep3B and Hep3B IFN-γ cells expressed GPC3, whereas only Hep3B IFN-γ cells showed high expression of PD-L1, which further validated that IFN-γ could induce PD-L1 expression in Hep3B cells. We normalized antigen expression by tumor-specific GFP. In comparison with the CD19 control, no GPC3 loss was found in the tumor treated with GPC3 CAR. The inducible expression of PD-L1 in tumors treated with PD-L1 CAR or bispecific CAR was higher than that in CD19 tumors, consistent with what we found in the cell co-culture system. Higher expression levels of both antigens, GPC3 and PD-L1, were found in the tumor treated with Combo compared with bispecific treatment. These results suggest that a combination of GPC3 CAR-T cells and PD-L1 CAR-T cells, but not bispecific CAR, synergistically killed Hep3B tumors.

DISCUSSION

The checkpoint molecule PD-L1 is highly expressed on many tumors in a constitutive or IFN-γ-inducible manner. IFN-γ is the critical functional cytokine released from effector T cells; however, the increased expression of PD-L1 in tumor cells binding to PD-1 in effector T cells results in T cell exhaustion and inhibition of T cell functions.[30] We hypothesized that development of CAR-T cells targeting PD-L1 could kill solid tumors by recognizing constitutive or inducible expression of PD-L1 in the immunosuppressive TME. To test our hypothesis, we isolated a panel of anti-PD-L1 single-domain antibodies from a newly established, semi-synthetic nurse shark V_{NAR} library. The best candidate, V_{NAR} B2, showed a specific binding ability to PD-L1 and was cross-reactive with human and mouse antigens. Significantly, B2 functionally blocked the interaction between PD-L1 and PD-1. We found that the single-domain-based CAR fragment could be highly expressed in human PBMCs, indicating that single-domain antibodies are suitable to be engineered into the CAR format because they are smaller, easily expressible, and more stable.

Although several shark V_{NAR}s showed a promising effect in academic and pre-clinical disease therapy research,[31–33] the potential immunogenicity of V_{NAR}s might limit their clinical development, including CAR-T cell therapy. Current FDA-approved CD19-targeted CAR-T cell therapies use murine-derived scFv (FMC63) and develop humoral and/or cellular immune responses against scFv in some individuals as one of the tumor relapse mechanisms.[34] Kovalenko et al.[35] humanized the framework of V_{NAR} by grafting antigen-binding domains to a human framework and observed that it largely retained antigen-binding specificity and affinity compared with the parental V_{NAR}. To address this issue, we would next humanize the B2 V_{NAR} and evaluate its immunogenicity in the CAR-T format. To test the efficacy of CAR (B2) T cells *in vivo*, we established an orthotopic xenograft mouse model bearing human breast cancer. We observed significantly effective tumor inhibition and metastasis prevention caused by CAR (B2) T cells, but there was still a modest tumor recurrence at week 5 after treatment. We did not find antigen (PD-L1) loss in tumor cells, and we thought the tumor recurrence was because (1) five million

Figure 5. CAR (B2) T cells lysed Hep3B tumor by targeting inducible PD-L1 and improved CAR-T efficacy in bispecific CAR and combination manners

(A) Inducible PD-L1 expression in Hep3B cells upon 50 μg/mL IFN-γ stimulation followed by depletion of IFN-γ at 24 h. (B) Inducible PD-L1 expression in the Hep3B cells after 24-h incubation with CAR (B2) T cells at an E/T ratio of 1:2. Shown are IFN-γ levels in cell supernatants of CAR (CD19) T cells or CAR (B2) T cells co-cultured with Hep3B cells. (C) Schematic of the Hep3B xenograft NSG model infused i.p. with five million CAR (B2) T cells and CAR (CD19) T cells after 12 days of tumor inoculation. (D) Representative bioluminescence image of Hep3B tumor growth in the xenograft model. (E) Tumor bioluminescence growth curve. (F) Inducible PD-L1 expression in Hep3B cells co-cultured with GPC3 CAR-T cells at an E/T ratio of 1:2 or 1:1 for 24 h. (G) Strategy of bispecific CAR-T cells and combination CAR-T cells targeting GPC3 and PD-L1. (H) Cytolytic activity of CAR-T cells on Hep3B cells after 24- or 72-h incubation *in vitro*. (I) TNF-α, IL-2, and IFN-γ concentration in the co-culture supernatant from (H) as measured by ELISA. Values represent mean ± SEM. $^{**}p < 0.01$; $^{***}p < 0.001$; $^{****}p < 0.0001$.

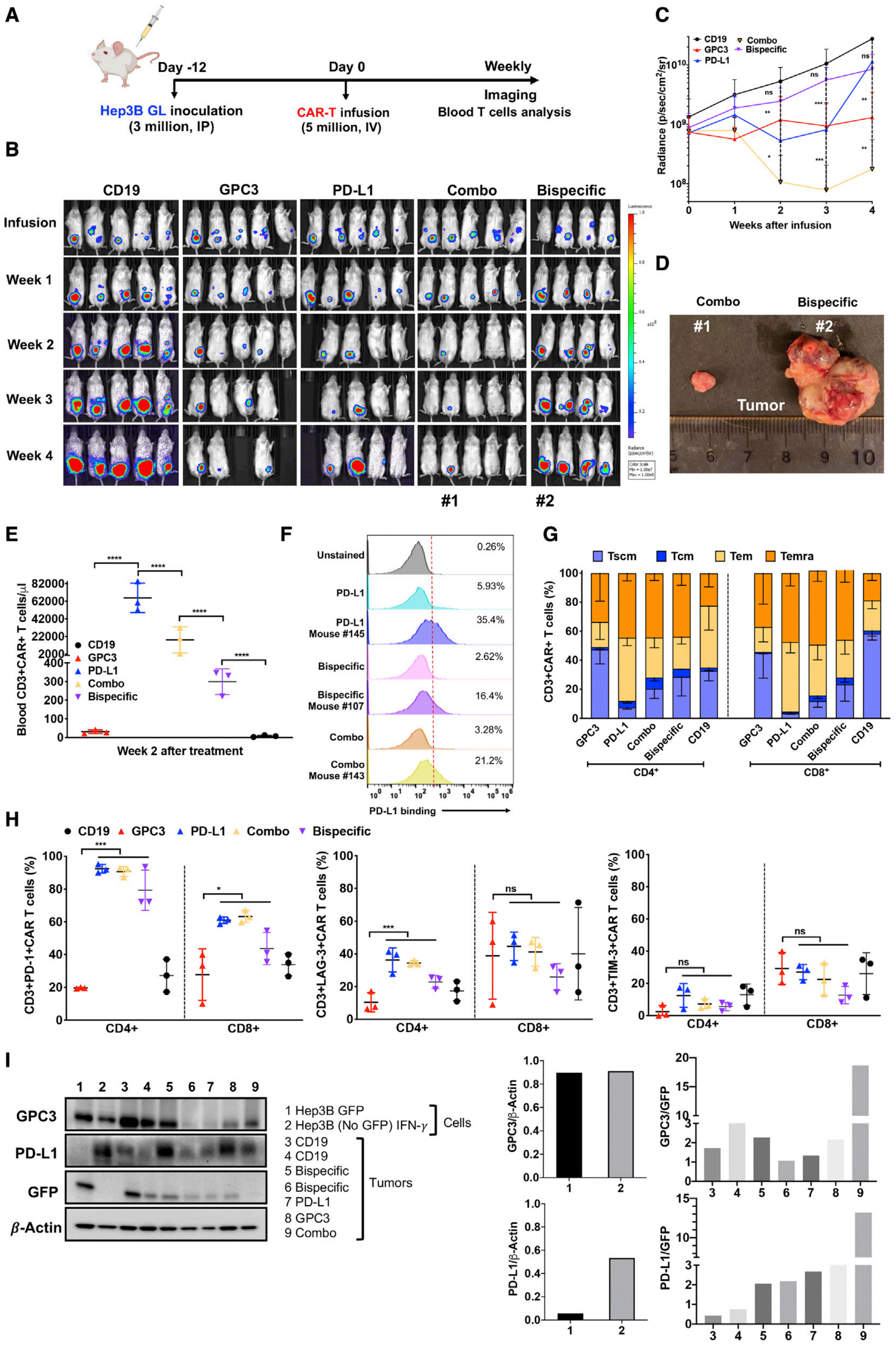

(legend on next page)

CAR-T cells might not be enough to cause tumor-free, (2) the long-persistent CAR-T cells were partially exhausted because of increased expression of PD-1 (Figure 6E).

PD-L1 is not only overexpressed in a more significant number of malignancies but also in immune cells in the TME.[13] T cells express low levels of endogenous PD-L1, which leads to development of CAR-T cells that target PD-L1 is somewhat intricate by killing PD-L1-expressing tumor cells and blocking the PD-1/PD-L1 checkpoint axis.[36,37] To address the potential toxicity of anti-PD-L1 CAR-T cells, a previous study tested CAR-T cells targeting mPD-L1 in a syngeneic mouse colon adenocarcinoma model using immunocompetent C57BL/6 mice.[17] These CAR-T cells increased mouse survival without apparent side effects. Antigen exposure of CAR-T cells may lead to T cell fratricide and exhaustion, impairing the proliferation and persistence of CAR-T cells *in vitro* and *in vivo*. Xie et al.[17] reported that camelid V_HH-based anti-mPD-L1 CAR-T cells can “self-activate” *in vitro*, and PD-L1 deficient CAR-T cells can live longer than WT CAR-T cells. However, during 7–12 days of *in vitro* co-culture with CD3/CD28 microbeads, we did not find upregulation of PD-L1 (Figure S3) or exhaustion markers (PD-1, TIM-3, and LAG-3) in activated CAR (B2) T cells compared with mock T cells. These events were probably due to PD-L1 antigen endocytosis caused by anti-PD-L1 CAR-T cells themselves.[37,38] We did not observe any cytolytic phenomena or upregulated IFN-γ expression in cultured CAR (B2) T cells, indicating that the cytotoxicity of CAR (B2) T cells was not triggered by the T cells’ endogenous PD-L1. We consider that less tonic signaling of our shark V_{NAR}-based CAR (B2) T cells may be due to the relatively low binding affinity of B2 V_{NAR}. Ghorashian et al.[39] reported enhanced proliferation and anti-tumor activity in a lower-affinity CD19 CAR compared with that in clinical high-affinity CAR (CD19) T cells, indicating that the increased immunoreceptor affinity may adversely affect T cell responses.

The combination strategy might be more feasible in solid tumor therapy to overcome tumor escape mechanisms and enhance the anti-tumor effect of CAR-T cells, such as combining CAR-T cells with monoclonal antibodies, small molecules, or bispecific CAR-T cells targeting different tumor-specific antigens.[40,41] In our study, we found that CAR (B2) T cells could kill liver cancer cells by targeting inducible PD-L1 in the immunosuppressive TME (Figure 5D), whereas B2 V_{NAR} did not show a significant benefit in improving the cytotoxicity of CAR (GPC3) T cells even though it functionally blocked the interaction of PD-1 with PD-L1 (Figure S4). Thus, we constructed a bispecific CAR-T cell targeting the HCC tumor-specific antigen GPC3 and the inducible tumor-immunosuppressive antigen PD-L1. Surprisingly, bispecific CAR-T cells worked best *in vitro* but only slightly inhibited liver tumor progression *in vivo* and performed even worse than individual GPC3 CAR-T cells and PD-L1 CAR-T cells.

We may optimize the bispecific CAR construct in future work by engineering two CAR fragments into one construct.[19] Combination of GPC3 CAR-T cells and PD-L1 CAR-T cells achieved a synergistic anti-tumor effect *in vivo*. A previous study reported that combination of anti-mesothelin CAR-T cells with PD-L1 CAR-T cells did not repress tumor growth synergistically in patient-derived xenograft (PDX) because PD-L1 CAR-T killed mesothelin CAR-T cells by targeting its endogenous PD-L1 antigen.[37] We did not observe upregulated PD-L1 expression in GPC3 CAR-T cells, probably because of different CAR constructs. On the other hand, we found that expansion of the CAR-T cell count in mouse blood is highly correlated with the PD-L1 CAR construct, and this may be due to cross-recognition of B2 V_{NAR} with endogenous mouse antigen. However, CAR-T cell treatment mice were healthy and did not experience body weight loss, indicating that our PD-L1 CAR-T cells are safe in mice. Although PD-1 was highly expressed in blood-recovered CAR (B2) T cells (Figure 6G), the CAR (B2) T cells recovered from mouse spleens still efficiently lysed MDA-MB-231 tumor cells (Figure 4G), probably because of B2 V_{NAR} blocking the interaction of PD-1 with PD-L1, although not entirely.

Our results demonstrate the feasibility and efficacy of CAR-T cells targeting the tumor immunosuppressive microenvironment antigen PD-L1 against aggressive solid tumors. To improve treatment of solid tumors, future efforts should be directed at utilizing genome editing to develop “off-the-shelf,” fratricide-resistant, PD-L1-targeted CAR-T cells lacking endogenous PD-L1 and the T cell receptor α chain.

MATERIALS AND METHODS

Construction of a synthetic 18-aa CDR3 nurse shark V_{NAR} phage library

We constructed the new synthetic 18-aa CDR3 nurse shark V_{NAR} phage library based on our previous naive nurse shark library.[26] For the V_{NAR}s DNA cassettes, a non-canonical cysteine in CDR1

Figure 6. Combination of PD-L1 CAR-T cells and GPC3 CAR-T cells achieved a synergistic anti-tumor effect *in vivo*

(A) Schematic of the Hep3B xenograft NSG model infused i.p. with an equivalent of a total of five million CAR-T cells after 12 days of tumor inoculation. (B) Representative bioluminescence image of Hep3B tumor growth in the xenograft model. (C) Tumor bioluminescence growth curve. (D) At the end of the study, the sizes of tumors in mice from the combination CAR group (mouse 1 mouse) and bispecific group (mouse 2). (E) Absolute CAR-T cell count detected in mouse peripheral blood after 2 weeks of treatment and absolute CAR-T concentration (cells per microliter) ±SD for all evaluated mice in each treatment group. (F) The binding ability of CAR T cells recovered *in vitro* and *in vivo* (2 weeks after treatment) to PD-L1 antigen using flow cytometry. (G) The relative proportion of stem cell-like memory (T_{SCM}), central memory (T_{CM}), effector memory (T_{EM}), and terminally differentiated effector memory (T_{EMRA}) cell subsets defined by CD62L, CD45RA, and CD95 expression in $CD4^+$ and $CD8^+$ CAR^+ T cell population in mouse blood at week 2 of treatment. (H) Exhaustion marker expression on $CD4^+$ and $CD8^+$ CAR^+ T cell populations in mouse blood at week 2 after treatment. (I) Western blotting detects GPC3 and PD-L1 expression in Hep3B tumor xenografts. The expression of GPC3 and PD-L1 in Hep3B GFP (no. 1) and Hep3B (no GFP) IFN-γ cells (no. 2) was normalized by β-actin. The expresssion of GPC3 and PD-L1 in tumor samples (no. 3–9) was nomalized by tumor-specific GFP expression.

was mutated to tyrosine (C29Y) using the naive shark library V_{NAR} pComb3x plasmid as the template. Subsequently, a pair of randomized 18-aa CDR3 primers was designed to amplify the CDR3 loop using the PCR method. PCR products were circularized by intra-molecular self-ligation in 1 mL of ligation buffer using T4 DNA ligase (New England Biolabs, Ipswich, MA). Finally, the ligation products were purified by removing the enzymes and transformed into 500 μL of electroporation-competent TG1 cells (Lucigen, Middleton, WI) to make the phage library.

Phage panning

The phage panning protocol has been described previously.[26,42] The mPD-L1 protein bought from R&D Systems was used for four rounds of panning. Details are provided in the supplemental information.

Affinity binding and blocking activity

The binding kinetics of the V_{NAR}-hFc (produced by GenScript) to hPD-L1-His protein (SinoBiological) was determined using the Octet RED96 system (FortéBio) at the Biophysics Core (National Heart, Lung, and Blood Institute [NHLBI]) as described previously.[43] The blocking activity of B2-hFc was determined using the BLI Octet platform as described previously[44] and sandwich ELISA. We provide details in the supplemental information.

Generation of anti-PD-L1 V_{NAR}-based CAR-T cells

We generated the PD-L1-target, shark V_{NAR}-based, CAR-T cell lentiviral vector following the design principle of a CAR construct published in our previous study.[10] Briefly, the V_{NAR} fragment of B2 was subcloned into a CAR construct (pMH330). The CAR-expressing lentivirus was produced as described previously.[10] Whole blood was collected from healthy donors under the Oklahoma Blood Institute Institutional Review Board approval. Human peripheral blood mononuclear cells (PBMCs) isolated from healthy donors were stimulated for 24 h using anti-CD3/anti-CD28 antibody-coated beads (Invitrogen) at a bead:cell ratio of 2:1 according to the manufacturer's instructions in the presence of IL-2.

In vitro cytolysis of CAR-T cells and activation assays

The cytotoxicity of CAR-T cells was determined by a luciferase-based assay. In brief, luciferase-expressing MDA-MB-231 and Hep3B tumor cells were used to establish a cytolytic assay. Cytolysis of PD-L1-targeted CAR (B2) T cells was detected by co-culture with MDA-MB-231 GL and Hep3B GL at various E/T ratios for 24 or 96 h, followed by measurement of luciferase activity using a luciferase assay system (Promega) on Victor (PerkinElmer). Supernatants were collected for TNF-α, IL-2, and IFN-γ detection using an ELISA kit (BD Biosciences). In the killing blocking assay of CAR-T cells, varying concentrations of soluble B2 protein were added to tumor CAR-T cells incubated for 24 and 48 h.

Animal studies

5-week-old female NSG mice (NCI, Frederick, MD) were housed and treated under a protocol (LMB-059) approved by the Institutional Animal Care and Use Committee of the NIH. A total of three million MDA-MB-231 GL cells were suspended in a mixture of PBS:Matrigel (BD Biosciences) at 1:1 and inoculated into the inguinal mammary fat pad to establish the orthotopic MDA-MB-231 model. The peritoneal Hep3B xenograft tumor model was established as described previously.[10] Tumor volume was calculated as $^1/_2$ (length × width^2) and bioluminescence intensity (Xenogen IVIS Lumina). When the average tumor size reached the indicated size, five million CAR-T cells were injected i.v. into mouse models. *Ex vivo* T cells were isolated from mouse spleens using a Miltenyi Biotec tumor dissociation kit and cultured *in vitro* with 40 ng/μL IL-2, IL-7, and IL-21 in the culture medium.

Statistical analysis

All experiments were repeated at least three times to ensure reproducibility of results. All statistical analyses were performed using GraphPad Prism and are presented as mean ± SEM. Results were analyzed using 2-tailed unpaired Student's t-test. A p value of less than 0.05 was considered statistically significant.

SUPPLEMENTAL INFORMATION

Supplemental information can be found online at https://doi.org/10.1016/j.omto.2022.02.015.

ACKNOWLEDGMENTS

This work was supported by the Intramural Research Program of the NIH, the National Cancer Institute (NCI), the Center for Cancer Research (CCR) FLEX Program Synergy Award (to G.M. and M.H.), and the NCI CCR FLEX Program Technology Development Award (to M.H.). We thank the NCI CCR Animal Resource Program/NCI Biological Testing Branch, NCI CCR/Leidos Animal Facility, Grzegorz Piszczek, and Di Wu of the NHLBI Biophysics Core, NCI CCR Flow Cytometry Core Facility for assisting with animal support, V_{NAR} single-domain antibody kinetics/affinity analysis, and cellular staining, respectively. We thank Alan Hoofring (NIH Medical Arts) for creating the graphical abstract and the NIH Fellows Editorial Board for editorial assistance. The anti-PD-L1 shark V_{NAR} single-domain antibodies, including B2, are the subject of pending patent applications assigned to the NIH and are available for license in certain fields of use to qualified candidates. Please contact M.H. (NCI) at homi@mail.nih.gov if you are interested in pursuing a license.

AUTHOR CONTRIBUTIONS

Conception and design, D.L., G.M., and M.H.; development of methodology, D.L., H.E., G.M., C.-P.D., and M.H.; acquisition of data, D.L., H.E., J.H., and T.L.; analysis and interpretation of data, D.L., H.E., J.H., and T.L.; writing, D.L. and M.H.; review and/or revision of manuscript, T.L., G.M., C.-P.D., and M.H.; study supervision, M.H. All authors read and approved the final manuscript.

DECLARATION OF INTERESTS

M.H., G.M., D.L., H.E., and C.-P.D. are inventors on US provisional patent application no. 63/208,755 assigned to the NIH "Cross Species Single Domain Antibodies Targeting PD-L1 For Treating Solid Tumors."

REFERENCES

1. Rosenberg, S.A., Restifo, N.P., Yang, J.C., Morgan, R.A., and Dudley, M.E. (2008). Adoptive cell transfer: a clinical path to effective cancer immunotherapy. Nat. Rev. Cancer *8*, 299–308. https://doi.org/10.1038/nrc2355.

2. Rosenberg, S.A., and Restifo, N.P. (2015). Adoptive cell transfer as personalized immunotherapy for human cancer. Science *348*, 62–68. https://doi.org/10.1126/science.aaa4967.

3. Kochenderfer, J.N., Wilson, W.H., Janik, J.E., Dudley, M.E., Stetler-Stevenson, M., Feldman, S.A., Maric, I., Raffeld, M., Nathan, D.A., Lanier, B.J., et al. (2010). Eradication of B-lineage cells and regression of lymphoma in a patient treated with autologous T cells genetically engineered to recognize CD19. Blood *116*, 4099–4102. https://doi.org/10.1182/blood-2010-04-281931.

4. Maher, J., Brentjens, R.J., Gunset, G., Riviere, I., and Sadelain, M. (2002). Human T-lymphocyte cytotoxicity and proliferation directed by a single chimeric TCRzeta/CD28 receptor. Nat. Biotechnol. *20*, 70–75. https://doi.org/10.1038/nbt0102-70.

5. Imai, C., Mihara, K., Andreansky, M., Nicholson, I.C., Pui, C.H., Geiger, T.L., and Campana, D. (2004). Chimeric receptors with 4-1BB signaling capacity provoke potent cytotoxicity against acute lymphoblastic leukemia. Leukemia *18*, 676–684. https://doi.org/10.1038/sj.leu.2403302.

6. Song, D.G., Ye, Q., Carpenito, C., Poussin, M., Wang, L.P., Ji, C., Figini, M., June, C.H., Coukos, G., and Powell, D.J., Jr. (2011). *In vivo* persistence, tumor localization, and antitumor activity of CAR-engineered T cells is enhanced by costimulatory signaling through CD137 (4-1BB). Cancer Res. *71*, 4617–4627. https://doi.org/10.1158/0008-5472.CAN-11-0422.

7. Gross, G., Waks, T., and Eshhar, Z. (1989). Expression of immunoglobulin-T-cell receptor chimeric molecules as functional receptors with antibody-type specificity. Proc. Natl. Acad. Sci. U S A *86*, 10024–10028. https://doi.org/10.1073/pnas.86.24.10024.

8. Kochenderfer, J.N., and Rosenberg, S.A. (2013). Treating B-cell cancer with T cells expressing anti-CD19 chimeric antigen receptors. Nat. Rev. Clin. Oncol. *10*, 267–276. https://doi.org/10.1038/nrclinonc.2013.46.

9. Li, N., Fu, H., Hewitt, S.M., Dimitrov, D.S., and Ho, M. (2017). Therapeutically targeting glypican-2 via single-domain antibody-based chimeric antigen receptors and immunotoxins in neuroblastoma. Proc. Natl. Acad. Sci. U S A *114*, e6623–e6631. https://doi.org/10.1073/pnas.1706055114.

10. Li, D., Li, N., Zhang, Y.F., Fu, H., Feng, M., Schneider, D., Su, L., Wu, X., Zhou, J., Mackay, S., et al. (2020). Persistent polyfunctional chimeric antigen receptor T Cells that target glypican 3 eliminate orthotopic hepatocellular carcinomas in mice. Gastroenterology *158*, 2250–2265. https://doi.org/10.1053/j.gastro.2020.02.011.

11. Lv, J., Zhao, R.C., Wu, D., Zheng, D.W., Wu, Z.P., Shi, J.X., Wei, X.R., Wu, Q.T., Long, Y.G., Lin, S.M., et al. (2019). Mesothelin is a target of chimeric antigen receptor T cells for treating gastric cancer. J. Hematol. Oncol. *12*, 18. https://doi.org/10.1186/s13045-019-0704-y.

12. Zhang, Z., Jiang, D., Yang, H., He, Z., Liu, X., Qin, W., Li, L., Wang, C., Li, Y., Li, H., et al. (2019). Modified CAR T cells targeting membrane-proximal epitope of mesothelin enhances the antitumor function against large solid tumor. Cell Death Dis. *10*, 476. https://doi.org/10.1038/s41419-019-1711-1.

13. Sun, C., Mezzadra, R., and Schumacher, T.N. (2018). Regulation and function of the PD-L1 checkpoint. Immunity *48*, 434–452. https://doi.org/10.1016/j.immuni.2018.03.014.

14. Dong, H., Strome, S.E., Salomao, D.R., Tamura, H., Hirano, F., Flies, D.B., Roche, P.C., Lu, J., Zhu, G., Tamada, K., et al. (2002). Tumor-associated B7-H1 promotes T-cell apoptosis: a potential mechanism of immune evasion. Nat. Med. *8*, 793–800. https://doi.org/10.1038/nm730.

15. Weinstock, M., and McDermott, D. (2015). Targeting PD-1/PD-L1 in the treatment of metastatic renal cell carcinoma. Ther. Adv. Urol. *7*, 365–377. https://doi.org/10.1177/1756287215597647.

16. Sznol, M. (2014). Blockade of the B7-H1/PD-1 pathway as a basis for combination anticancer therapy. Cancer J. *20*, 290–295. https://doi.org/10.1097/PPO.0000000000000056.

17. Xie, Y.J., Dougan, M., Jailkhani, N., Ingram, J., Fang, T., Kummer, L., Momin, N., Pishesha, N., Rickelt, S., Hynes, R.O., and Ploegh, H. (2019). Nanobody-based CAR T cells that target the tumor microenvironment inhibit the growth of solid tumors in immunocompetent mice. Proc. Natl. Acad. Sci. U S A *116*, 7624–7631. https://doi.org/10.1073/pnas.1817147116.

18. Fabian, K.P., Padget, M.R., Donahue, R.N., Solocinski, K., Robbins, Y., Allen, C.T., Lee, J.H., Rabizadeh, S., Soon-Shiong, P., Schlom, J., and Hodge, J.W. (2020). PD-L1 targeting high-affinity NK (t-haNK) cells induce direct antitumor effects and target suppressive MDSC populations. J. Immunother Cancer *8*, e000450. https://doi.org/10.1136/jitc-2019-000450.

19. Zhao, W., Jia, L., Zhang, M., Huang, X., Qian, P., Tang, Q., Zhu, J., and Feng, Z. (2019). The killing effect of novel bi-specific Trop2/PD-L1 CAR-T cell targeted gastric cancer. Am. J. Cancer Res. *9*, 1846–1856.

20. Chailyan, A., Marcatili, P., and Tramontano, A. (2011). The association of heavy and light chain variable domains in antibodies: implications for antigen specificity. FEBS J. *278*, 2858–2866. https://doi.org/10.1111/j.1742-4658.2011.08207.x.

21. Hamers-Casterman, C., Atarhouch, T., Muyldermans, S., Robinson, G., Hamers, C., Songa, E.B., Bendahman, N., and Hamers, R. (1993). Naturally occurring antibodies devoid of light chains. Nature *363*, 446–448. https://doi.org/10.1038/363446a0.

22. Flajnik, M.F., and Kasahara, M. (2010). Origin and evolution of the adaptive immune system: genetic events and selective pressures. Nat. Rev. Genet. *11*, 47–59. https://doi.org/10.1038/nrg2703.

23. Muyldermans, S. (2013). Nanobodies: natural single-domain antibodies. Annu. Rev. Biochem. *82*, 775–797. https://doi.org/10.1146/annurev-biochem-063011-092449.

24. Criscitiello, M.F., Saltis, M., and Flajnik, M.F. (2006). An evolutionarily mobile antigen receptor variable region gene: doubly rearranging NAR-TcR genes in sharks. Proc. Natl. Acad. Sci. U S A *103*, 5036–5041. https://doi.org/10.1073/pnas.0507074103.

25. English, H., Hong, J., and Ho, M. (2020). Ancient species offers contemporary therapeutics: an update on shark VNAR single domain antibody sequences, phage libraries and potential clinical applications. Antib. Ther. *3*, 1–9. https://doi.org/10.1093/abt/tbaa001.

26. Feng, M., Bian, H., Wu, X., Fu, T., Fu, Y., Hong, J., Fleming, B.D., Flajnik, M.F., and Ho, M. (2019). Construction and next-generation sequencing analysis of a large phage-displayed VNAR single-domain antibody library from six naive nurse sharks. Antib. Ther. *2*, 1–11. https://doi.org/10.1093/abt/tby011.

27. Matz, H., and Dooley, H. (2019). Shark IgNAR-derived binding domains as potential diagnostic and therapeutic agents. Dev. Comp. Immunol. *90*, 100–107. https://doi.org/10.1016/j.dci.2018.09.007.

28. Ubah, O.C., Buschhaus, M.J., Ferguson, L., Kovaleva, M., Steven, J., Porter, A.J., and Barelle, C.J. (2018). Next-generation flexible formats of VNAR domains expand the drug platform's utility and developability. Biochem. Soc. Trans. *46*, 1559–1565. https://doi.org/10.1042/BST20180177.

29. Camacho-Villegas, T., Mata-Gonzalez, T., Paniagua-Solis, J., Sanchez, E., and Licea, A. (2013). Human TNF cytokine neutralization with a vNAR from Heterodontus francisci shark: a potential therapeutic use. MAbs *5*, 80–85. https://doi.org/10.4161/mabs.22593.

30. Chen, L.P., and Han, X. (2015). Anti-PD-1/PD-L1 therapy of human cancer: past, present, and future. J. Clin. Invest. *125*, 3384–3391. https://doi.org/10.1172/Jci80011.

31. Kovaleva, M., Johnson, K., Steven, J., Barelle, C.J., and Porter, A. (2017). Therapeutic potential of shark anti-ICOSL VNAR domains is exemplified in a murine model of autoimmune non-infectious uveitis. Front. Immunol. *8*, 1121. https://doi.org/10.3389/fimmu.2017.01121.

32. Camacho-Villegas, T.A., Mata-Gonzalez, M.T., Garcia-Ubbelohd, W., Nunez-Garcia, L., Elosua, C., Paniagua-Solis, J.F., and Licea-Navarro, A.F. (2018). Intraocular penetration of a vNAR: *in vivo* and *in vitro* VEGF165 neutralization. Mar. Drugs *16*, 113. https://doi.org/10.3390/md16040113.

33. Hasler, J., Flajnik, M.F., Williams, G., Walsh, F.S., and Rutkowski, J.L. (2016). VNAR single-domain antibodies specific for BAFF inhibit B cell development by molecular mimicry. Mol. Immunol. *75*, 28–37. https://doi.org/10.1016/j.molimm.2016.05.009.

34. Heng, G., Jia, J., Li, S., Fu, G., Wang, M., Qin, D., Li, Y., Pei, L., Tian, X., Zhang, J., et al. (2020). Sustained therapeutic efficacy of humanized anti-CD19 chimeric antigen receptor T Cells in relapsed/refractory acute lymphoblastic leukemia. Clin. Cancer Res. *26*, 1606–1615. https://doi.org/10.1158/1078-0432.Ccr-19-1339.

35. Kovalenko, O.V., Olland, A., Piche-Nicholas, N., Godbole, A., King, D., Svenson, K., Calabro, V., Muller, M.R., Barelle, C.J., Somers, W., et al. (2013). Atypical antigen recognition mode of a shark immunoglobulin new antigen receptor (IgNAR) variable domain characterized by humanization and structural analysis. J. Biol. Chem. *288*, 17408–17419. https://doi.org/10.1074/jbc.M112.435289.

36. Xie, Y.J., Dougan, M., Jailkhani, N., Ingram, J., Fang, T., Kummer, L., Momin, N., Pishesha, N., Rickelt, S., Hynes, R.O., and Ploegh, H. (2019). Nanobody-based CAR T cells that target the tumor microenvironment inhibit the growth of solid tumors in immunocompetent mice (vol 116, pg 7624, 2019). Proc. Natl. Acad. Sci. U S A *116*, 16656. https://doi.org/10.1073/pnas.1912487116.

37. Qin, L., Zhao, R.C., Chen, D.M., Wei, X.R., Wu, Q.T., Long, Y.G., Jiang, Z.W., Li, Y.Q., Wu, H.P., Zhang, X.C., et al. (2020). Chimeric antigen receptor T cells targeting PD-L1 suppress tumor growth. Biomark. Res. *8*, 19. https://doi.org/10.1186/s40364-020-00198-0.

38. Hamieh, M., Dobrin, A., Cabriolu, A., van der Stegen, S.J.C., Giavridis, T., Mansilla-Soto, J., Eyquem, J., Zhao, Z., Whitlock, B.M., Miele, M.M., et al. (2019). CAR T cell trogocytosis and cooperative killing regulate tumour antigen escape. Nature *568*, 112–116. https://doi.org/10.1038/s41586-019-1054-1.

39. Ghorashian, S., Kramer, A.M., Onuoha, S., Wright, G., Bartram, J., Richardson, R., Albon, S.J., Casanovas-Company, J., Castro, F., Popova, B., et al. (2019). Enhanced CAR T cell expansion and prolonged persistence in pediatric patients with ALL treated with a low-affinity CD19 CAR. Nat. Med. *25*, 1408–1414. https://doi.org/10.1038/s41591-019-0549-5.

40. Pan, Z., Di, S., Shi, B., Jiang, H., Shi, Z., Liu, Y., Wang, Y., Luo, H., Yu, M., Wu, X., and Li, Z. (2018). Increased antitumor activities of glypican-3-specific chimeric antigen receptor-modified T cells by coexpression of a soluble PD1-CH3 fusion protein. Cancer Immunol. Immunother. *67*, 1621–1634. https://doi.org/10.1007/s00262-018-2221-1.

41. Hegde, M., Mukherjee, M., Grada, Z., Pignata, A., Landi, D., Navai, S.A., Wakefield, A., Fousek, K., Bielamowicz, K., Chow, K.K., et al. (2016). Tandem CAR T cells targeting HER2 and IL13Ralpha2 mitigate tumor antigen escape. J. Clin. Invest. *126*, 3036–3052. https://doi.org/10.1172/JCI83416.

42. Ho, M., Kreitman, R.J., Onda, M., and Pastan, I. (2005). In vitro antibody evolution targeting germline hot spots to increase activity of an anti-CD22 immunotoxin. J. Biol. Chem. *280*, 607–617. https://doi.org/10.1074/jbc.M409783200.

43. Maus, M.V., and June, C.H. (2016). Making better chimeric antigen receptors for adoptive T-cell therapy. Clin. Cancer Res. *22*, 1875–1884. https://doi.org/10.1158/1078-0432.CCR-15-1433.

44. Petersen, R.L. (2017). Strategies using bio-layer interferometry biosensor technology for vaccine research and development. Biosensors (Basel) *7*, 49. https://doi.org/10.3390/bios7040049.

OMTO, Volume *24*

Supplemental information

A novel PD-L1-targeted shark V_{NAR} single-domain-based CAR-T cell strategy for treating breast cancer and liver cancer

Dan Li, Hejiao English, Jessica Hong, Tianyuzhou Liang, Glenn Merlino, Chi-Ping Day, and Mitchell Ho

Supplementary materials

Cell culture

We obtained human breast cancer cell line MDA-MB-231, human ovarian cancer (OC) cells lines IGROV-1 and NCI-ADR-RES, human pancreatic cancer (PDAC) cell lines KLM1, Panc-1, and SU8686, and lung cancer cell line H522 from American Type Culture Collection (ATCC). OVCAR8 (ovarian cancer) and EKVX (non-small cell lung cancer, or NSCLC) were obtained from National Cancer Institute (Development Therapeutics Program). Steven M. Albelda provided L55 (NSCLC) at the University of Pennsylvania (Philadelphia, PA). MDA-MB-231 was transduced with a lentiviral vector encoding a GFP-firefly-luciferase (GFP-Luc) following our protocol.[1] PD-L1 knockout (KO) MDA-MB-231 cell line was constructed in the present study using clustered regularly interspaced short palindromic repeats (CRISPR)/CRISPR-associated protein 9 (CRISPR-Cas9) method. We generated the construct following the design principle as described previously.[2] Briefly, two single-guide RNAs (sgRNAs) targeted endogenous PD-L1 promotor (predicted from the EPD database) were designed and used to subclone into a LentiCRISPRv2 vector (Addgene plasmid #52961) and sorted to generate single clones by flow cytometry. Glenn Merlino (NCI) provided the murine melanoma cell line B8979HC (induced in an HGF-tg;CDKN2A$^{fl/fl}$;Tyr-CreERT2-tg mouse by UV and tamoxifen) and the canine tumor cell line Jones. Hep3B was obtained from ATCC, and Hep3B overexpressed GFP-Luc cell line was established in a previous study.[3] MDA-MB-231 and Hep3B cells were cultured in DMEM supplemented with 10% FBS, 1% L-glutamine, and 1% penicillin–streptomycin; other cell lines mentioned above were cultured in RPMI. Cells were maintained in a humidified atmosphere containing 5% CO_2 at 37°C.

Phage panning

We conducted phage panning following our laboratory protocol.[4,5] The Nunc 96-well Maxisorp plate (Thermo Scientific) was coated with 100 μg/ml mPD-L1 in PBS overnight at 4°C. Subsequently, the plate was blocked with 2% bovine serum albumin in PBS for 1 hour at room temperature. Then 10^{10}-10^{11} CFU

of pre-blocked phage supernatant in blocking buffer was added to each well for 1 hour at room temperature to allow binding. After four rounds of panning, the enriched bound phages were eluted with 100 μl pH 2.0 elution buffer at room temperature after four washes with PBS containing 0.05% Tween-20. The eluate was neutralized with 30 μl of 1 M Tris-HCl buffer (pH 8.5) and was used to infect freshly prepared *E. coli* TG1 cells. Single colonies were picked after four rounds of panning and identified by phage ELISA.

Antibody production and purification

The soluble antibody protein-his-flag was produced and purified as previously described.[6] Briefly, the coding sequences of PD-L1 specific V_{NAR}s in the pComb3x phagemids were transformed into HB2151 *E.coli* cells. The colonies were pooled for culture in 2 L 2YT media containing 2% glucose, 100 μg/ml ampicillin at 37°C until the OD600 reached 0.8–1. Culture media was then replaced with 2YT media containing 1mM IPTG (Sigma), 100 μg/ml ampicillin, and was shaken at 30°C overnight for soluble protein production. The bacteria pellet was spun down and lysed with polymyxin B (Sigma) for 1 h at 37°C to release the soluble protein. The supernatant was harvested after lysis, and purified using the HisTrap column (Cytiva/GE Healthcare) using AKTA.

Affinity binding and blocking activity

All experiments were performed on an Octet instrument (ForteBio) at 30°C and reagents were prepared in 0.5% BSA, 0.1% Tween20 PBS, pH 7.4 buffer. hPD-L1-his protein was immobilized onto Ni-NTA sensor tips at 5 μg/ml for 120 s. The antigen-coated tips are then dipped into the buffer to stabilize the curve and subsequently dipped into 100 nM or 50 nM B2-hFc or F5-hFc for association and dissociation measurements for a time window of 180 s and 300 s. To detect the blocking activity of B2-hFc, hPD-L1-his protein was firstly loaded onto the Ni-NTA sensor tips. Subsequently, the sensor tips were dipped into wells containing 500 nM of V_{NAR}-hFc for 300 s, followed by 500 nM hPD-1-hFc protein (Sino Biological)

for 300 s, and lastly 300 s of dissociation in the buffer. Raw data was processed using Octet Data Analysis Software 9.0.

ELISA

The phage ELISA was performed as previously described[6]. Briefly, the Nunc 96-well Maxisorp plate was coated with 5 μg/ml antigenic proteins, including mPD-L1-his, mPD-L1-hFc, hPD-L1-his, hPD-L1-hFc, and the irrelevant antigen human IgG and PBS in 50 μl PBS per well overnight at 4°C. The plate was blocked with 2% BSA for 1 hour at room temperature. Pre-blocked phage supernatant was then added to the plate. The binding activity was determined with horseradish peroxidase (HRP)-conjugated mouse anti-M13 antibody (GE Healthcare). To test whether anti-PD-L1 V_{NAR}s can block the interaction of hPD-1 and hPD-L1, hPD-1-hFc was coated on 96-well Maxisorp plate overnight at 4°C. Coated wells were blocked with 2% BSA. After washing, the wells were incubated hPD-L1, 2 μg/ml V_{NAR}-hFc (or PBS as control), and then HRP-conjugated anti-his antibody for detection. To detect whether a shark B2 V_{NAR} is specific to human PD-L1, and not B7-H3, the antigenic proteins hPD-L1 and hB7-H3 were coated onto the 96-well Maxisorp plate. PD-L1 specific binder B2 and B7-H3 specific binder were used to measure the binding affinity and specificity to PD-L1 and B7-H3.

To predict the binding epitope of anti-PD-L1 shark V_{NAR}s, we designed total 24 peptides based on the hPD-L1 extracellular domain (ECD) amino acid sequence. Each synthesized peptide (produced by GenScript) was 18 AA in length with 9 AA overlapped, indicating the minimum antigenic region of hPD-L1 ECD recognized by the V_{NAR}s can be narrowed down by step-by-step peptide mapping onto a 9-mer peptide epitope. The ELISA was performed in this assay. In brief, a total of 24 peptides were coated onto the 96-well Maxisorp plate. 5 μg/ml B2-his-flag, A11-his-flag, and F5-his-flag were then added followed with HRP-conjugated anti-flag antibody. The binding activity was determined by OD_{450}. Experiments were performed in triplicate and repeated three times with similar results.

Flow cytometry

Surface PD-L1 expression was detected by anti-PD-L1 monoclonal antibody (Biolegend) and goat-anti-human IgG-PE (Jackson ImmunoResearch). Tumor cells were incubated with 10 µg/ml of each V_{NAR}-his-flag, followed by incubation with mouse anti-flag conjugated with allophycocyanin (APC) (Jackson ImmunoResearch). 50 µg/ml IFN-γ was used to induce PD-L1 expression in Hep3B tumor cells *in vitro*. The transduction efficiency of CAR (B2) T cells was detected by surface anti-EGFR human monoclonal antibody cetuximab (Erbitux) and goat-anti-human IgG conjugated with APC. 10 µg/ml recombinant PD-L1-hFc protein, GPC3-hFc, and goat-anti-human IgG conjugated with APC were used to test antigen-binding ability of CAR-T cells. T cell exhaustion was evaluated via PE PD-1, PE TIM-3, and PE LAG-3 (Thermo Fisher Scientific). 100 µl blood was collected from mice and 1X RBC lysis buffer (eBiosciences) was used to remove red blood cells. To determine the absolute number of CAR-T cells in mouse blood, BV711 CD3, Erbitux, and goat-anti-human IgG conjugated with Alexa Fluor 488 CD8 (Biolegend) were used to stain CD3+CAR+ T cells. Counting Beads 123count™ eBeads was used for counting the absolute number of cells. T cell immunophenotyping was performed by surface staining with antibodies against the following antigens: APC-H7 CD4, BV605 CD8, BV421 CD45RA, APC CD62L (BD Bioscience), and PE CD95 (Biolegend). Data acquisition was performed using SONY SA3800 (Sony Biotechnology) and analyzed using FloJo software (Tree Star).

Western blot

Cells were lysed with ice-cold lysis buffer (Cell Signaling Technology), and total protein was isolated by centrifugation at 10,000g for 10 minutes at 4°C. Protein concentration was measured using a Bicinchoninic acid assay (Pierce) by the manufacturer's specifications. For each cell lysates of 20 µg, they were loaded onto a 4-20% SDS-PAGE gel for electrophoresis. Total protein was extracted from *in vivo* tumors derived from MDA-MB-231 or Hep3B mice. 10 µg cell lysates were used to detect the expression of PD-L1, GPC3, GFP, and β-Actin. The antibodies, including anti-human PDL1 mAb, anti-human GAPDH mAb, anti-GFP

mAb, and anti-human β-Actin mAb were obtained from Cell Signaling Technology. YP7 mAb, which our lab previously produced, was used to test GPC3 expression.[7]

References

1. Feng, M., Zhang, J., Anver, M., Hassan, R., and Ho, M. (2011). In vivo imaging of human malignant mesothelioma grown orthotopically in the peritoneal cavity of nude mice. Journal of Cancer *2*, 123-131. 10.7150/jca.2.123.
2. Li, N., Wei, L., Liu, X., Bai, H., Ye, Y., Li, D., Li, N., Baxa, U., Wang, Q., Lv, L., Chen, Y., et al. (2019). A Frizzled-Like Cysteine-Rich Domain in Glypican-3 Mediates Wnt Binding and Regulates Hepatocellular Carcinoma Tumor Growth in Mice. Hepatology *70*, 1231-1245. 10.1002/hep.30646.
3. Li, D., Li, N., Zhang, Y.-F., Fu, H., Feng, M., Schneider, D., Su, L., Wu, X., Zhou, J., Mackay, S., Kramer, J., et al. (2020). Persistent Polyfunctional Chimeric Antigen Receptor T Cells That Target Glypican 3 Eliminate Orthotopic Hepatocellular Carcinomas in Mice. Gastroenterology. https://doi.org/10.1053/j.gastro.2020.02.011.
4. Ho, M., Kreitman, R.J., Onda, M., and Pastan, I. (2005). In vitro antibody evolution targeting germline hot spots to increase activity of an anti-CD22 immunotoxin. J Biol Chem *280*, 607-617. 10.1074/jbc.M409783200.
5. Kim, H., and Ho, M. (2018). Isolation of Antibodies to Heparan Sulfate on Glypicans by Phage Display. Curr Protoc Protein Sci *94*, e66. 10.1002/cpps.66.
6. Feng, M., Bian, H., Wu, X., Fu, T., Fu, Y., Hong, J., Fleming, B.D., Flajnik, M.F., and Ho, M. (2019). Construction and next-generation sequencing analysis of a large phage-displayed VNAR single-domain antibody library from six naive nurse sharks. Antibody therapeutics *2*, 1-11. 10.1093/abt/tby011.
7. Phung, Y., Gao, W., Man, Y.G., Nagata, S., and Ho, M. (2012). High-affinity monoclonal antibodies to cell surface tumor antigen glypican-3 generated through a combination of peptide immunization and flow cytometry screening. mAbs *4*, 592-599. 10.4161/mabs.20933.

Supplementary Figures

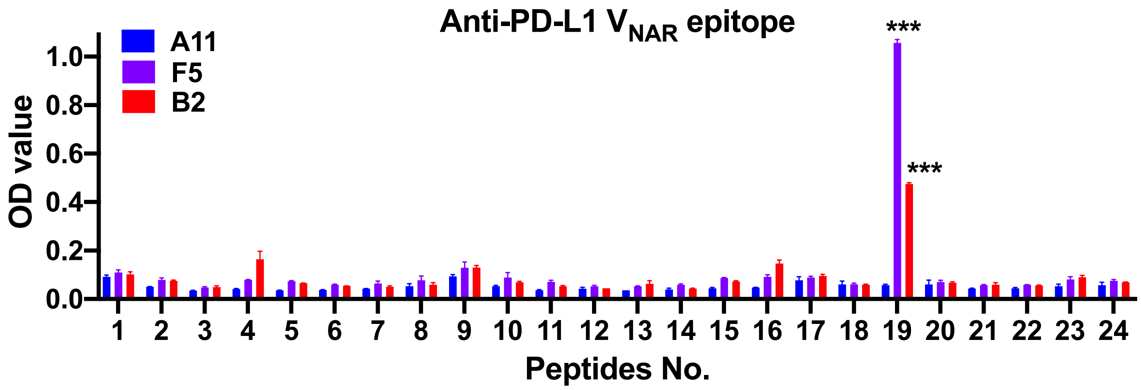

Supplemental Figure 1. Epitope mapping of individual anti-PD-L1 V_{NAR}s using truncation peptide arrays detected by ELISA. Total 24 peptides were designed based on hPD-L1 ECD. Each peptide is 18 amino acid in length and overlapped 9 AA with adjacent peptide.

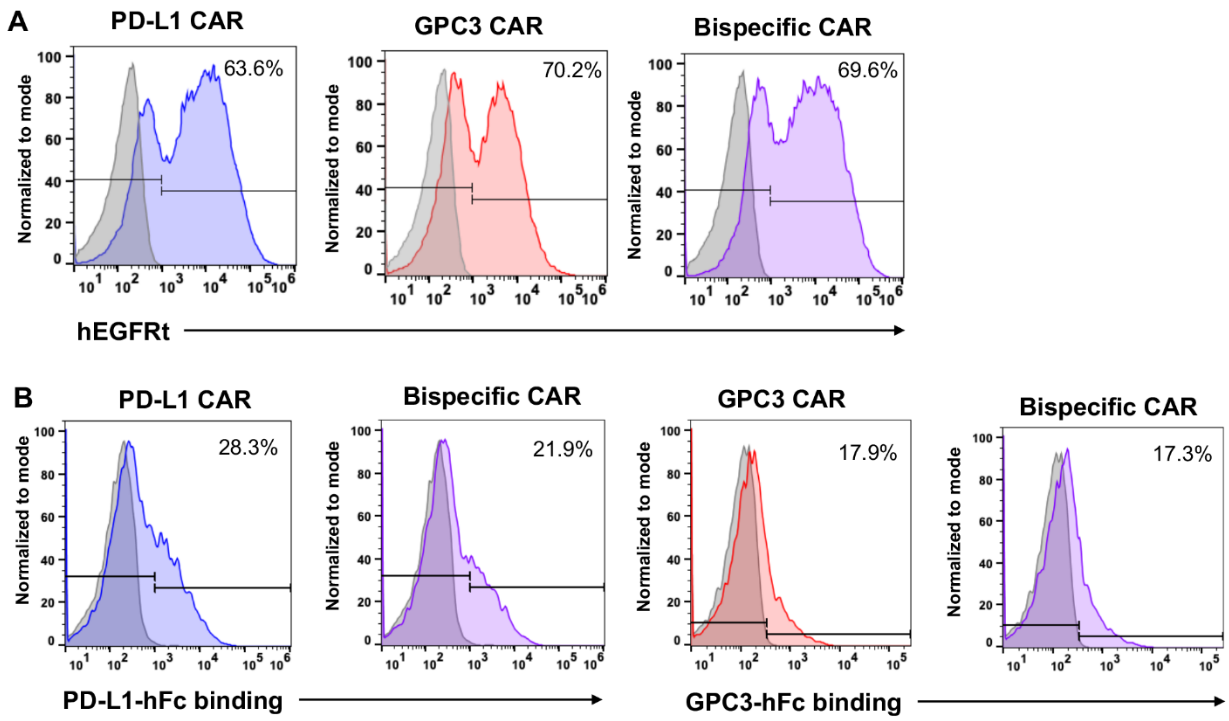

Supplemental Figure 2. CAR expression and antigen-binding ability of PD-L1 CAR (B2) T, GPC3 CAR-T, and bispecific CAR-T cells. (A) The CAR expression in T cells was detected by flow cytometry based on hEGFRt expression. (B) The antigen-binding (PD-L1-hFc and GPC3-hFc) ability of PD-L1 CAR (B2) T, GPC3 CAR-T, and bispecific CAR-T cells.

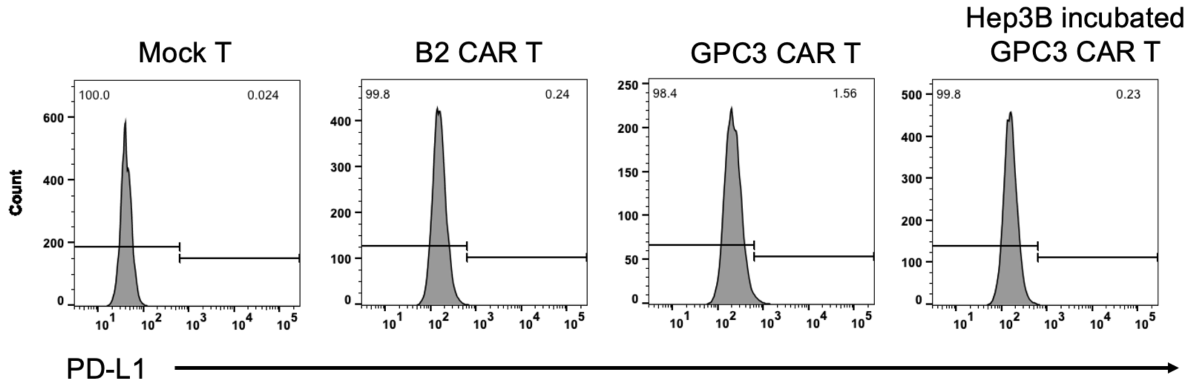

Supplemental Figure 3. PD-L1 expression in mock T, CAR (B2) T, GPC3 CAR-T, and Hep3B tumor incubated GPC3 CAR-T cells.

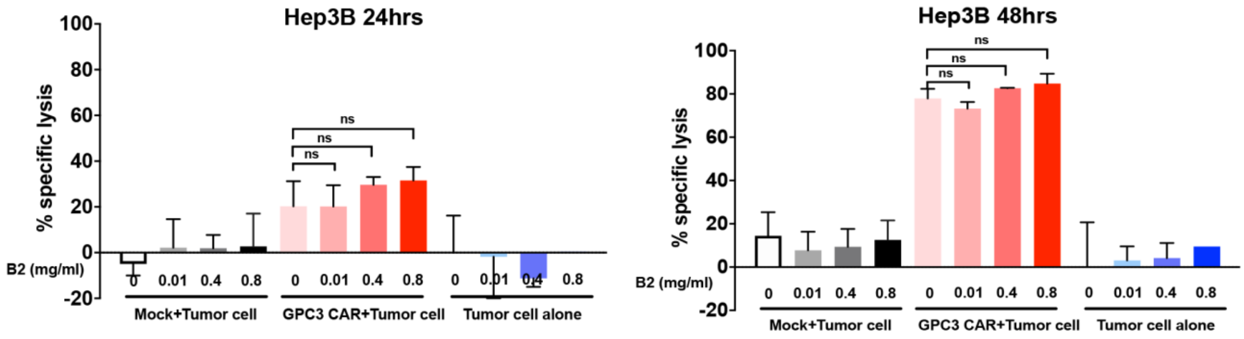

Supplemental Figure 4. Monovalent B2 nanobody did not improve GPC3 CAR-T cells killing on Hep3B cells after 24 hours or 48 hours of incubation. Tumor cells alone or mock T cells incubation in the presence of B2 were used as the control in this study. Statistical analyses are shown from three independent experiments. Values represent mean ± SEM. $^{**}P < .01$, $^{***}P < .001$, $^{****}P < .0001$, ns, not significant.
